# A comprehensive evaluation of rodent malaria parasite genomes and gene expression

**DOI:** 10.1186/s12915-014-0086-0

**Published:** 2014-10-30

**Authors:** Thomas D Otto, Ulrike Böhme, Andrew P Jackson, Martin Hunt, Blandine Franke-Fayard, Wieteke A M Hoeijmakers, Agnieszka A Religa, Lauren Robertson, Mandy Sanders, Solabomi A Ogun, Deirdre Cunningham, Annette Erhart, Oliver Billker, Shahid M Khan, Hendrik G Stunnenberg, Jean Langhorne, Anthony A Holder, Andrew P Waters, Chris I Newbold, Arnab Pain, Matthew Berriman, Chris J Janse

**Affiliations:** Wellcome Trust Sanger Institute, Hinxton, Cambridge UK; Department of Infection Biology, Institute of Infection and Global Health, University of Liverpool, Liverpool, UK; Leiden Malaria Research Group, Department of Parasitology, Leiden University Medical Center, Leiden, The Netherlands; Department of Molecular Biology, Science faculty, Radboud Institute for Molecular Life Sciences, Radboud University, Nijmegen, The Netherlands; Institute of Infection, Immunity & Inflammation, School of Medical, Veterinary & Life Sciences, & Wellcome Centre for Molecular Parasitology, Glasgow Biomedical Research Centre, University of Glasgow, Glasgow, Scotland UK; Division of Parasitology, MRC National Institute for Medical Research, Mill Hill, London UK; Unit of Malariology, Institute of Tropical Medicine, Antwerp, Belgium; Weatherall Institute of Molecular Medicine, University of Oxford, John Radcliffe Hospital, Oxford, UK; Weatherall Institute of Molecular Medicine, John Radcliffe Hospital, Headington, Oxford UK; Biological and Environmental Sciences and Engineering (BESE) Division, King Abdullah University of Science and Technology (KAUST), Thuwal, Kingdom of Saudi Arabia

**Keywords:** *Plasmodium chabaudi*, *Plasmodium berghei*, *Plasmodium yoelii*, Genomes, RNA-seq, Genotypic diversity, Multigene families, *pir*s, Phylogeny

## Abstract

**Background:**

Rodent malaria parasites (RMP) are used extensively as models of human malaria. Draft RMP genomes have been published for *Plasmodium yoelii*, *P. berghei* ANKA (*Pb*A) and *P. chabaudi* AS (*Pc*AS). Although availability of these genomes made a significant impact on recent malaria research, these genomes were highly fragmented and were annotated with little manual curation. The fragmented nature of the genomes has hampered genome wide analysis of *Plasmodium* gene regulation and function.

**Results:**

We have greatly improved the genome assemblies of *Pb*A and *Pc*AS, newly sequenced the virulent parasite *P. yoelii* YM genome, sequenced additional RMP isolates/lines and have characterized genotypic diversity within RMP species. We have produced RNA-seq data and utilised it to improve gene-model prediction and to provide quantitative, genome-wide, data on gene expression. Comparison of the RMP genomes with the genome of the human malaria parasite *P. falciparum* and RNA-seq mapping permitted gene annotation at base-pair resolution. Full-length chromosomal annotation permitted a comprehensive classification of all subtelomeric multigene families including the ‘*Plasmodium* interspersed repeat genes’ (*pir*). Phylogenetic classification of the *pir* family, combined with *pir* expression patterns, indicates functional diversification within this family.

**Conclusions:**

Complete RMP genomes, RNA-seq and genotypic diversity data are excellent and important resources for gene-function and post-genomic analyses and to better interrogate *Plasmodium* biology. Genotypic diversity between *P. chabaudi* isolates makes this species an excellent parasite to study genotype-phenotype relationships. The improved classification of multigene families will enhance studies on the role of (variant) exported proteins in virulence and immune evasion/modulation.

**Electronic supplementary material:**

The online version of this article (doi:10.1186/s12915-014-0086-0) contains supplementary material, which is available to authorized users.

## Background

Rodent malaria parasites (RMP) are used extensively as models of human malaria [1,2]. Four different species that infect African rodents have been adapted for laboratory use: *Plasmodium berghei*, *P. yoelii*, *P. chabaudi* and *P. vinckei.* Small differences exist in the biology of the different RMP in laboratory mice and this makes them particularly attractive models to investigate different aspects of human malaria. Specifically, *P. chabaudi* is a model to investigate mechanisms of drug resistances and immune evasion, in particular antigenic variation [3,4]. It invades normocytes and reticulocytes and mostly produces chronic, non-lethal, infections. In contrast, *P. berghei* preferentially invades reticulocytes and usually produces infections in mice that induce severe pathology [2]. In combination with different mouse strains it has been used as a model to study immunopathology, experimental cerebral malaria, pregnancy-associated malaria and lung pathology [2]. *P. yoelii* is widely used in studies on the biology of liver stages and on innate and acquired immunity against liver stages [5,6]. Blood stage *P. yoelii* parasites of some lines are restricted to reticulocytes whereas others can invade all red blood cells and have been used to study receptors for erythrocyte binding [7,8]. The availability of efficient reverse genetics technologies for *P. berghei* and *P. yoelii* [9-11] and the ability to analyse these parasites throughout the complete life cycle have made these two species the preferred models for analysis of *Plasmodium* gene function [12-14]. For these two species more than 600 different genetically modified mutants have been reported [15].

The first draft RMP genome was published in 2002 for *P. yoelii yoelii* 17XNL [16]. This was followed by publication of draft genomes of *P. berghei* ANKA (*Pb*A) and *P. chabaudi chabaudi* AS (*Pc*AS) in 2005 [17]. Comparisons with the genome of the human parasite *P. falciparum* and other primate malaria species defined a large set of core genes that are shared between RMPs and primate malarias [18-20]. Although availability of draft RMP genomes made a significant impact in applying post-genomic technologies for understanding malaria biology [18] and were used in many follow-up functional genomics studies to analyse gene regulation and function [9,10], these RMP genomes were highly fragmented and were annotated with little or no manual curation. The fragmented nature of the genomes has hampered genome wide analysis of gene regulation and function, especially of the (subtelomeric) multigene families. To utilise RMP models to their full potential, we therefore undertook production of high quality reference genomes: for *PbA* and *PcAS* large-scale improvement of their existing genomes, with re-sequencing, re-analysis and manual re-annotation, and for *P. y. yoelii* a genome sequence was produced *de novo* from the virulent YM line using the latest sequencing technologies and computational algorithms. In addition, we have utilised comprehensive RNA-seq data derived from a number of life-cycle stages to both improve gene model prediction and to provide genome-wide, quantitative data on gene expression. By sequencing additional isolates/lines of *P. berghei, P. yoelii* and *P. chabaudi* (including the subspecies *P. c. adami*) we have documented genotypic diversity that exists within different RMP species. The availability of RMP reference genomes in combination with the RNA-seq and genotypic diversity data will serve as excellent resources for gene-function and post-genomic analyses and, therefore, better interrogation of *Plasmodium* biology and development of anti-malaria interventions.

The genomes of RMP contain a number of multigene families located in the subtelomeric chromosomal regions. These include a large family of so-called ‘*Plasmodium* interspersed repeat genes’ (*pir*) [16], that are present also in other human/primate *Plasmodium* species [20-23]. Most of these gene families are expressed in blood stages and these proteins show features that have been reported to contribute to immune evasion through antigenic variation [24-26] and may play a role in the sequestration of infected red blood cells and virulence [26,27]. As a result of the improved annotation, we have been able to define all multigene families in the RMP genomes. Comparative phylogenetic analyses of the *pir* genes and analyses of *pir* expression patterns in blood stages of *P. berghei* provide evidence of functional diversification within this gene family. The improved classification of multigene families will enhance studies on the role of (variant) exported proteins in virulence and evasion and modulation of the immune system.

## Results

### Generation of high-quality RMP reference genomes

With a combination of Sanger and second generation sequencing (that is, Illumina and 454), automated scaffolding, gap closure, error correction and annotation transfer, followed by manual inspection, we obtained highly accurate and almost complete reference genomes of *Pb*A, *Pc*AS and *P. y. yoelii* YM (*Py*YM). This resulted in a significant reduction in contig number for the *Pb*A and *Pc*AS genomes (Table 1) compared with existing highly fragmented drafts [16,17]. The new assemblies contain 4,979, 5,139 and 5,675 protein-coding genes for *Pb*A, *Pc*AS and *Py*YM, respectively, with 487 and 409 novel genes in the genomes of *Pb*A and *Pc*AS that were absent in the draft genomes (Table 1, Additional file 1). More than 98% of the predicted genes are now present as full-length gene models and we were able to ascribe putative functions (that is, they are not annotated as encoding hypothetical proteins of unknown function) to 56% to 61% of these. This percentage is comparable to the 60% of the *P. falciparum* genes that have annotated functions. As a result of eliminating incomplete gene models and merging multiple incorrect gene models into single gene models and by removing mouse DNA sequence contamination, only 63% and 77% of the previously annotated *Pb*A and *Pc*AS genes were mapped back to the new genomes. The RMP reference genomes have a size of 18.5 to 21.9 Mb (Table 1), confirming the smaller genome sizes of RMPs compared with primate malaria species but both the mitochondrial and apicoplast RMP genomes are highly comparable in size and gene content to those of *P. falciparum* (Table 1). The predicted proteomes were analysed for the presence of PEXEL-motifs, a characteristic of host-exported proteins, using ExportPred v2.0 [28]. Between 97 and 119 PEXEL-positive proteins were predicted for the different RMP. This indicates that, like *P. berghei*, the other RMP also contain three times more PEXEL-positive proteins than was previously predicted [29] (see Additional file 2).

**Table 1:** **Features of the reference genomes of** ***P. berghei*** **ANKA,** ***P. c. chabaudi*** **AS and** ***P. y. yoelii*** **YM**

**Genome features**	***P. berghei*** **ANKA**		***P. c. chabaudi*** **AS**		***P. y. yoelii*** **YM**	***P. falciparum 3D7*** ^**a**^
	**Previous assembly [** 17 **]**	**New assembly**	**Previous assembly [** 17 **]**	**New assembly**	**New assembly**	
**Nuclear genome**						
Genome size (Mb)	18.0	18.5	16.9	18.8	21.9	23.3
G + C content (%)	-	22.1	-	23.6	21.1	19.4
Chromosomes	14	14	14	14	14	14
Synteny breaks^b^	-	-	1	1	0	ND
Contigs	7497	220	10,679	40	195	14
Sequence coverage	4x	237x	4x	109x	627x	-
Genes^c^	5,864	4,979	5,698	5,139	5,675	5,419
Genes with functional annotation^d^	-	2,781 (56%)	-	2,927 (57%)	3,485 (61%)	3,234 (60%)
Novel genes (see Additional file 1)	-	487	-	409	-	-
**Mitochondrial genome**						
Genome size (bp)	-	5,957	-	5,949	6,512	5,967
G + C content (%)	-	30.9	-	30.9	30.7	31.6
Number of genes	-	3	-	3	3	3
**Apicoplast genome**						
Genome size (bp)	-	30,302	-	29,468	29,736	29,430
G + C content (%)	-	13.5	-	13.7	14.1	13.1
Genes	-	30	-	30	30	30

^a^Genome version: 1.5.2013; Apicoplast genome from accession numbers: X95275, X95276; ^b^compared to the PbA genome; ^c^in new versions, this includes pseudogenes and partial genes, but does not include non-coding RNA genes; ^d^figures include all genes except those annotated as ‘hypothetical’, ‘conserved Plasmodium protein, unknown function’, ‘conserved protein, unknown function’, ‘conserved rodent malaria protein, unknown function’ or ‘Plasmodium exported protein, unknown function’.

### Conserved chromosome organization and gene orthology between RMP and primate malaria parasites

The improved genomes confirmed the extensive conservation of the RMP genomes and the presence of only a single synteny breakpoint ([19]; Table 1). Despite the highly conserved internal regions of the 14 chromosomes, species-specific paralogous expansion and diversification of certain genes has occurred in each genome. Additional file 3A shows an example of such an expanded locus within a region of conserved synteny between *Pc*AS and *Py*YM. In *Pb*A only a single copy (PBANKA_091920) is present, whereas the genomes of *Pc*AS and *Py*YM contain multiple copies (*fam-d*; Table 2, Additional file 4), organized as a single gene cluster on chromosome 9.

**Table 2:** **Different (subtelomeric) multigene families in the RMP genomes**

**Gene family (new name)**	**Other (previous) names**	**Number of genes**								
			***Pb*** **A**			***Pc*** **AS**			***Py*** **YM**	
		**CG**	**PSG**	**FG**	**CG**	**PSG**	**FG**	**CG**	**PSG**	**FG**
***pir***	*pir, bir, cir, yir*	100	88	12	194	3	4	583	40	172
***RMP-fam-a***	*Pb-fam-1; Pc-fam-1; fam-a;* PYSTA	23	16	3	132	2	0	94	8	11
***RMP-fam-b***	*Pb-fam-3;* PYSTB	34	1	5	26	0	0	48	2	4
***RMP-fam-c***	PYSTC	6	0	-	10	0	-	22	0	-
***RMP-fam-d***	*Pc-fam*	1	0	-	17	4	-	5	0	-
***Early transcribed membrane protein***	*etramp*	7	-	-	13	-	-	12	-	-
***Reticulocyte binding protein, putative***	*P235; 235kDA protein*	6	-	8	8	-	0	11	-	3
	*rhoptry protein, putative*									
***Lysophospholipase***		4	1	-	28	0	-	11	1	-
***RMP-erythrocyte membrane antigen (RMP-EMA1)***	*pcema1*	1	0	0	13	1	1	1	0	0
***haloacid dehalogenase-like hydrolase, putative***		1	-	-	9	-	-	1	-	-
***‘Other subtelomeric genes’***		46	-	-	67	-	-	46	-	-

See Additional file 4 for details of all genes of *P. berghei* ANKA (*Pb*A), *P. c. chabaudi* AS (*Pc*AS) and *P. y. yoelii* YM (*Py*YM). CG: complete gene; FG: fragment; PSG: pseudogene.

**Figure 1:**
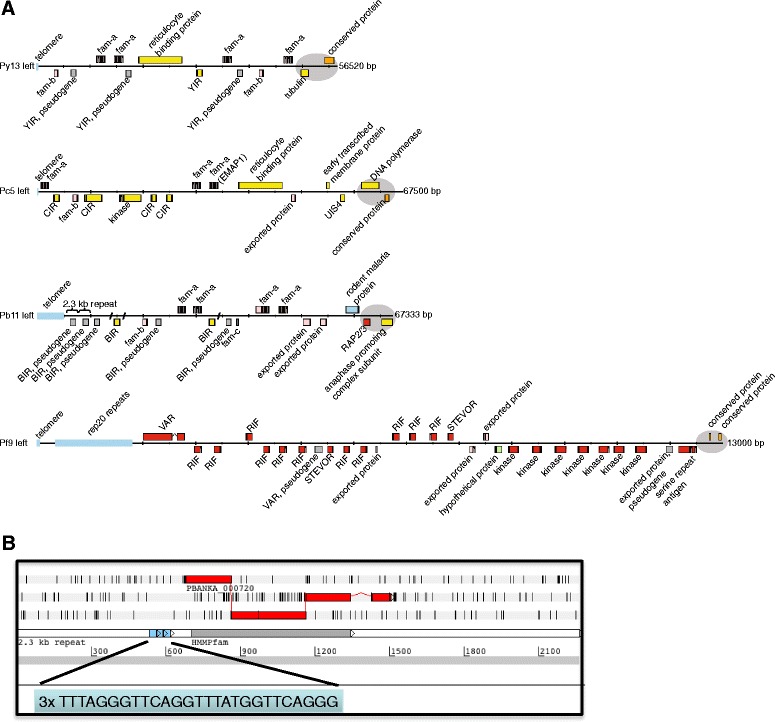
**Organization of subtelomeric regions of RMP chromosomes. A)** Organization of subtelomeric regions of chromosomes 13 of *Py*YM (left region), 5 of *Pc*AS (left region) and 11 of *Pb*A (left region). The order and orientation of the genes are shown, including genes belonging to the *pir, fam-a, fam-b* and *fam-c* gene families. Exons are shown in coloured boxes with introns as linking lines. As a comparison, a subtelomeric region of *P. falciparum* 3D7 chromosome 9 is shown. The shaded/grey areas mark the start of the conserved, syntenic regions. Black angular lines represent gaps. **B)** Artemis view showing a copy of the *Pb*A-specific 2.3 kb repeat element containing a fragmented and ‘pseudogenised’ *bir* gene (PBANKA_000720). Also shown is the location of three 27 bp telomeric repeat units. The HMM Pfam match ‘Cir_Bir_Yir PF06022’ (grey box) spans amino acid 6 to 239 with an e-value of 2.5e-77. RPM, rodent malaria parasites.

The re-annotation resulted in a better characterization of several non-coding and coding features of the chromosomes such as the centromeric and subtelomeric regions. As an example we show in Additional file 3B the size, location and GC-content of a positionally conserved centromere-containing region of chromosome 7 of *Pb*A and *Pc*AS. Figure 1A shows an example of the organization of RMP subtelomeric regions, visualizing the location of genes of multigene families. Although these regions contain members of multigene families that are shared between RMP, they are highly variable as a result of variation in gene copy number and the presence of species-specific genes and (non-coding) repeat sequences. For example, several *Pb*A subtelomeric regions contain many copies of a large, 2.3 kb, repeat element that is *P. berghei*-specific (Figure 1B; [30]). Based on the coverage-depth of Illumina sequence data mapped onto the 2.3 kb repeats, we estimated that the *Pb*A genome contains about 400 copies, representing approximately 4% of the total nucleotide content. In the *Pb*A genome only 47 of these repeats have been assembled and the remaining copies (approximately 350) are either located as clusters in the sequence gaps that still exist in the *Pb*A subtelomeric regions or are located within the existing current 2.3 kb repeat-arrays. These 2.3 kb repeats contain telomeric repeat sequences and many contain a copy of a highly degenerate *pir* pseudogene (Figure 1B; [30]) suggesting that the expansion of this repeat may have originally been driven by an expansion of *pir* gene numbers.

We compared all predicted RMP protein-coding genes with those of three primate malaria species, *P. falciparum*, *P. knowlesi* and *P. vivax* using OrthoMCL and divided the predicted RMP proteome into three different categories: (1) RMP proteins with orthologs in any of the primate malarias; (2) RMP-specific proteins with no orthologs in primate malarias; and (3) primate malaria-specific proteins with no orthologs in any of the RMP (see Additional file 5). Between the predicted RMP proteomes (15,793 proteins in total) and primate malaria proteomes (15,853 proteins in total), approximately 87% of the RMP proteins had detectable orthologs in at least one of the primate malarias and only 2,104 proteins (13.3%) were predicted to be RMP-specific. Of those 2,104 proteins, 1,854 (88.1%) are from gene families, as defined in Additional file 4. For 2,306 primate malaria proteins (14.6%) no orthologs have been detected in the RMP. Of these primate malaria specific genes, approximately 1,635 (70.9%) are subtelomeric genes or members of subtelomeric gene families (see Additional file 5).

### Genotypic diversity within RMP isolates: *P. chabaudi* isolates exhibit high level polymorphism amongst their genes

The availability of multiple isolates of RMP with different phenotypic traits offers the possibility of using genetics to study phenotype/genotype associations. To quantify the level of genotypic diversity across multiple RMP isolates we produced genome sequence data at a 79- to 437-fold coverage, from isolates of *P. berghei* (NK65, K173, SP11 and its pyrimethamine resistant descendant SP11 RLL), *P. c. chabaudi* (AJ, CB) and *P. c. adami* (DK, DS). In addition, we sequenced *P. y. yoelii* 17X (see Additional file 6 for parasite selection rationale and Additional file 7 for parasite origins). Single nucleotide polymorphisms (SNPs) in the genomes of these parasites were called by mapping the reads against their respective reference genomes (Table 3, Additional file 8) after excluding repetitive or low complexity regions of genes and members of multigene families (genes in Additional file 4). The level of polymorphism between the four *P. berghei* isolates is surprisingly low with SNPs detected in only 4 to 469 genes (Table 3, Additional file 8). In *P. berghei* the highest SNP numbers were found in two lines, SP11 RLL and K173cl1, which have been maintained in the laboratory for prolonged periods by mechanical blood passage between mice. Comparison of the genomes of SP11 and its pyrimethamine resistant descendant SP11 RLL, revealed the point mutation in the dihydrofolate reductase-thymidylate synthase gene, known to be involved in pyrimethamine resistance (see Additional file 8; [31]). Comparing the genomes of the non-lethal *P. y. yoelii* 17X isolate and its virulent descendant laboratory line YM showed limited polymorphism and revealed SNPs in the Duffy-binding protein that have been implicated in the different invasion and virulence phenotypes of these two lines (see Additional file 8; [7]). In contrast to the low numbers of *P. y yoelii* genes with SNPs (eight genes), large differences exist in gene copy number of subtelomeric multigene families (Table 3) which accounts for the difference in genome size between the two laboratory lines.

**Table 3:** **Sequence diversity and number of members of multigene families from different RMP isolates/lines**

**Isolate/line**	**Genome coverage**	**SNPs** ^**b**^	**Genes with SNPs** ^**b**^	**Assembly size (Mb)**	**Contigs**	***pir*** **genes** ^**c**^	***fam-a*** **genes** ^**c**^	***fam-b*** **genes** ^**c**^	***fam-c*** **genes** ^**c**^	***fam-d*** **genes** ^**c**^
***P. berghei*** **ANKA** ^**a**^	-	-	-	18.56	220	200	42	40	6	1
*P. berghei* NK65 E	437x	294	21	18.45	313	174	67	46	6	1
*P. berghei* NK65NY	161x	127	14	18.47	310	191	54	47	5	1
*P. berghei* SP11 A	268x	95	4	18.44	333	182	54	45	7	1
*P. berghei* SP11 RLL A	401x	2,098	345	18.32	275	170	58	50	6	1
*P. berghei* K173cl1	262x	2,759	469	18.72	123	224	54	41	7	1
***P. y. yoelii*** **YM** ^**a**^	-	-	-	22.03	195	795	113	54	22	5
*P. y. yoelii 17*X	289x	740	8	22.75	154	980	157	78	26	5
***P. c. chabaudi*** **AS** ^**a**^	-	-	-	18.83	40	201	134	26	10	21
*P. c. chabaudi* CB	79x	144,148	4,321	18.98	246	276	160	31	23	21
*P. c. chabaudi* AJ	127x	144,281	4,326	18.89	240	277	161	28	18	19
*P. c. adami* DS	98x	251,984	4,514	19.63	272	371	215	37	33	29
*P. c. adami* DK	248x	274,877	4,509	19.41	275	398	193	41	32	25

^a^Reference genomes (that is, *Pb*A, *Pc*AS and *Py*YM) to which sequence data from other isolates were mapped and analysed.; ^b^excluded from the analysis: all subtelomerically located genes (as mentioned in Additional file 4) and repetitive and low complexity regions of genes. Only single nucleotide polymorphisms (SNPs) were counted with at least 10 high quality mapped reads, 90% allele and 20% calls on each strand (see Additional file 8 for details of the SNPs in individual genes); ^c^including pseudogenes and fragments. RMP, rodent malaria parasites.

In contrast to the *P. berghei* isolates, the *P. chabaudi* isolates and subspecies have much higher SNP densities with 4,300 to 4,500 (out of 4,576) non-subtelomeric genes having at least one SNP (Table 3, Additional file 8). The high genotypic diversity is not only evident between the subspecies *P. c. chabaudi* and *P. c. adami*, but also between isolates of the same subspecies. For example, we found 94,668 and 71,074 unique SNPs (in 3,978 and 4,166 genes) in the *P. c adami* DK and DS isolates, respectively. Between different *P. chabaudi* isolates differences exist in virulence- and invasion phenotypes of blood stage infections (see Additional file 6). We detected multiple SNPs in *P. chabaudi* genes involved in binding to red blood cells (RBC) such as the Duffy-binding protein and reticulocyte binding proteins (see Additional file 8), genes that are associated with differences in virulence between *P. y. yoelii* lines*.* Isolate-specific protective immunity between *P. c. chabaudi* isolates has been linked to the merozoite surface protein 1 (MSP1; PCHAS_083130) [32,33]. Our analyses revealed an excess of non-synonymous substitutions (reflected in high Ka/Ks values) in *msp1* of all *P. chabaudi* isolates (see Additional file 8).

### High resolution, genome-wide expression data from different RMP life cycle stages

**Figure 2:**
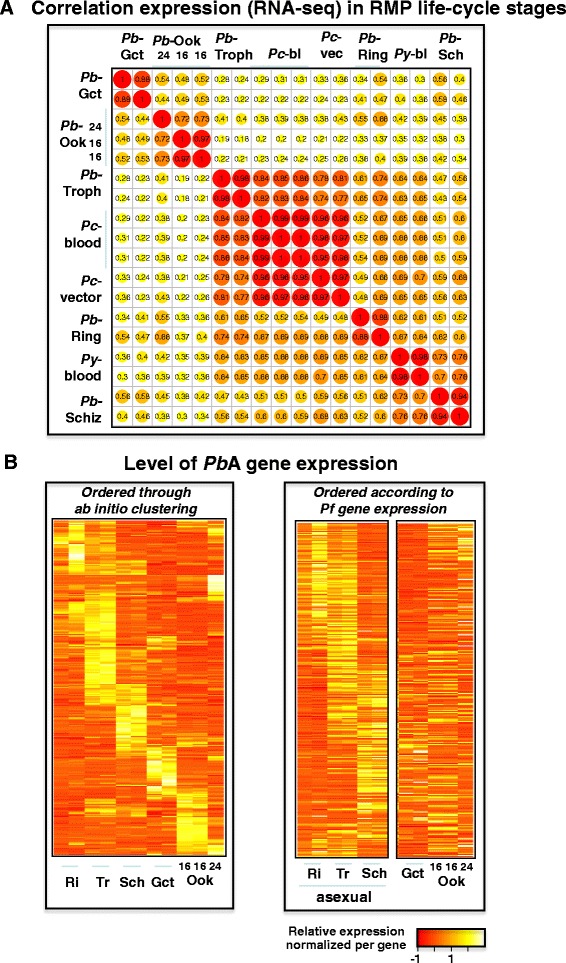
**Gene expression (RNAseq) in multiple RMP life cycle stages. A)** Spearman correlation of FPKM values of orthologous genes between life cycle stages of *Pb*A, *Pc*AS and *Py*YM. *Pb*A: ring (RI), trophozoite (Tr), schizont (Sch), gametocyte (Gct) and 16 and 24 hour ookinetes (Ook). *Pc*AS: trophozoites (trophozoites of blood (*Pc*-bl)-and vector-transmitted (*Pc*-vec) *Pc*AS; *Py*YM; blood stages (2 lines *Py*YM_WT and *Py*YM_MUT). **B)** Heat maps of expression (FPKM normalized by gene) of *Pb*A genes in different life cycle stages. Left panel, all *Pb*A genes ordered based on *P. berghei* expression pattern (FPKM values >21; in total 4,733 genes). Right panel, 2,236 *Pb*A genes with orthologs in P. *falciparum* and FPKM values >63, ordered according to the temporal expression levels (in asexual blood stages) of their *P. falciparum* orthologs as shown in [34]. FPKM, fragments per kilo base of exon per million fragments mapped; RMP, rodent malaria parasites.

To further improve gene annotation and to provide foundational data for gene-function studies, we generated RNA-seq data from several life-cycle stages. RNA was analysed from synchronised *Pb*A asexual blood stages (ring forms, late trophozoites and schizonts) and from purified gametocytes and ookinetes. In addition, RNA-seq data was generated from multiple samples of blood stage trophozoites of *Pc*AS and from blood stages of *Py*YM (see Additional file 9). To analyse the reproducibility of our RNA-seq data, we calculated Pearson correlations of the FPKM (fragments per kilo base of exon per million fragments mapped) values of RMP genes for which one-to-one ortholog relationships exist in the different RPM genomes (Figure 2A). Expression was highly correlated not only between biological replicates of the same species (r = 0.88 to 1.0), but also between comparable stages of different RMP, such as *Pb*A and *Pc*AS trophozoites (r = 0.77 to 0.86). Both the gametocyte and ookinete samples clustered separately from asexual blood stages, which reflects the different program of gene expression during sexual commitment and zygote development. Heat maps representing the expression of all *PbA* genes reveal clusters of genes with distinct expression patterns in the different life cycle stages (Figure 2B, left panel), consistent with both the morphological and functional differences between these stages. When *Pb*A genes are ordered according to the expression levels of their *P. falciparum* orthologs [34], ring-, trophozoite- and schizont-expressed genes display the expected characteristic temporal cascade of gene expression (Figure 2B, right panel). Genome wide expression data of developing ookinetes have not been published before. We found that mature (24 hour) ookinetes have a distinct expression pattern compared to immature, developing ookinetes (16 hour). In Additional file 10 an overview is presented of all genes that are up- or down regulated in the two developmental stages of ookinetes. Genome ontology (GO)-annotation of differentially regulated genes reveals that genes encoding proteins involved in protein phosphorylation, inner membrane and myosin complex formation and ATP binding are most significantly up-regulated in 16 hour ookinetes compared to 24 hour ookinetes (see Additional file 10). In contrast, mature ookinetes show a strong up-regulation of genes encoding proteins involved in protein translation and ribosome formation (see Additional file 10), most likely in preparation for the rapid growth expansion of the oocyst after ookinete traversal of the mosquito midgut wall.

To further improve the reference genomes we mapped the RNA-seq data onto the RMP genomes and visually inspected the alignments using the Artemis Comparison Tool (ACT), a genome viewing tool [35]. A comparative analysis with the *P. falciparum* 3D7 genome allowed us to determine gene structure at base-pair (bp) resolution for at least 89% of the genes. Of the 896 newly annotated protein-coding genes that were absent in the previous genome assemblies, 70% have primate malaria orthologs, 83% have expression evidence (RNA-seq FPKM values >21) and we could ascribe functions to 75% (see Additional file 1). The different RNA-seq data sets have also been used to confirm splice sites and to identify putative alternative splice sites (see Additional file 11). This analysis resulted in the identification of 839 alternative splicing events in a total of 567 RMP genes.

### Characterization of RMP multigene families

As a result of having dramatically improved the annotation of the subtelomeric regions we were able to accurately define the RMP multigene families that are located there (Table 2, Additional file 4). For proteins of nearly all of these families experimental evidence exists that they are exported into the host RBC in the absence of a PEXEL motif [29]. The *pir* family is the most abundant multigene family (see next section) encoding exported proteins that lack a canonical PEXEL motif. The second largest gene family is the *fam-a* gene family, formerly identified as the *pyst-a* family in *P. yoelii* 17XNL and named as *Pb-fam-1, Pc-fam-1 or fam-a* [16,17]. *Pb*A *fam-a* proteins are exported into the host RBC and can be transported to the RBC surface membrane [29] but lack a PEXEL-motif. Single copy orthologs have been defined in all primate malarias and the expansion of this family is RMP-specific. Most members have a subtelomeric location (see Additional files 12, 13 and 14), but all three RMP have at least one internally located copy that is positionally conserved with the primate malaria orthologs and, therefore, likely to represent the ancestral copy of this family. In order to standardise the naming of orthologous multigene families in different RMP, we have renamed the two multigene families, *pyst-b/pb-fam-3* and *pyst-c* genes [16,17,29] as *fam-b* and *fam-c*, respectively (Table 2; Additional file 4). The *fam-b* family is exclusively subtelomeric and is characterized by the presence of the *pyst-b* domain. Most members contain a transmembrane domain (58%), a signal peptide (75%) and PEXEL-motif (76%) (see Additional files 12, 13 and 14). *Pb*A *fam-b* proteins are exported into the host RBC [29]. The *fam-c* is also exclusively found in the subtelomeric regions and is characterized by the presence of a *pyst-c1* and/or *pyst-c2* domain [16]. Most members have a transmembrane domain (60%) and a signal peptide (92%) (see Additional files 12, 13 and 14) and only a small percentage (24%) contain a predicted PEXEL-motif.

Other subtelomeric multigene families include the ‘early transcribed family of proteins’ (ETRAMPs) and ‘putative reticulocyte binding proteins’ (Table 2, Additional files 4, 12, 13 and 14). ETRAMPS are small exported proteins with a predicted signal peptide and transmembrane domain but without a PEXEL-motif. These proteins are mainly located in the parasitophorous vacuole membrane [36,37]. The genes encoding putative reticulocyte binding proteins (RBP), that were first described in *P. yoelii* as Py235 and are expressed in merozoites [38], are clear orthologs of the reticulocyte binding proteins of *P. vivax* [39] and the RH proteins of *P. falciparum* [40]. These large proteins typically have a predicted signal sequence and at the C-terminus a transmembrane domain containing a rhomboid cleavage site and a cytoplasmic domain, although *P. falciparum* RH5 contains just the signal peptide and N-terminal ligand binding domain [41]. The RMPs have genes encoding two short RBPs reminiscent of *P. falciparum* RH5 (typified by PYYM_0101400 and PYYM_0701100) and six or more full length proteins (Table 2). Compared with *Py*YM, *Py*17X contains an additional full length RBP.

In *Pc*AS several other expanded gene families are present in the subtelomeric regions. These include ‘putative lysophospholipases’, ‘erythrocyte membrane antigen 1’ (EMA1), and ‘putative haloacid dehalogenase-like hydrolases’ (Table 2, Additional files 4, 12, 13 and 14). The genes encoding lysophospholipases are characterized by the ‘*pst-a’* domain [42] and all RMP have two copies with an internal chromosomal location that are syntenic with orthologs of primate malarias. For two of the five *Pb*A lysophospholipases evidence exists that they are exported into the RBC [29] and again they lack a PEXEL-motif. In *Pc*AS this family has expanded into 28 copies (Table 2, Additional file 4). In the genome of *Py*YM and *Pb*A only a single gene encoding EMA1 is present whereas *Pc*AS *ema1* has expanded to more than 10 copies in the subtelomeric regions (Table 2, Additional file 4). These PEXEL-negative proteins, first described in *P. chabaudi* [43] are associated with the RBC membrane. The gene encoding the putative haloacid dehalogenase-like hydrolase has expanded only in *Pc*AS, with eight subtelomeric copies.

A number of other genes are interspersed within the subtelomeric regions of RMP chromosomes. Many of these ‘other subtelomeric genes’ (46 to 67 genes; Table 2, Additional file 4) encode proteins that are RMP-specific and more than 96% of these proteins contain a predicted signal peptide, transmembrane domain or PEXEL-motif and for several proteins experimental evidence exists for their export into the host RBC cytoplasm. Combined, these observations indicate that most, if not all, RMP subtelomeric genes (apart from the RBP family) encode exported proteins and most lack a PEXEL-motif. The presence of large numbers of PEXEL-negative exported proteins in RMP indicates alternative export mechanisms possibly common to all *Plasmodium* species and investigations with highly tractable RMP species can, therefore, be used to understand these mechanisms better.

### The RMP pir multigene family: phylogeny and expression

**Figure 3:**
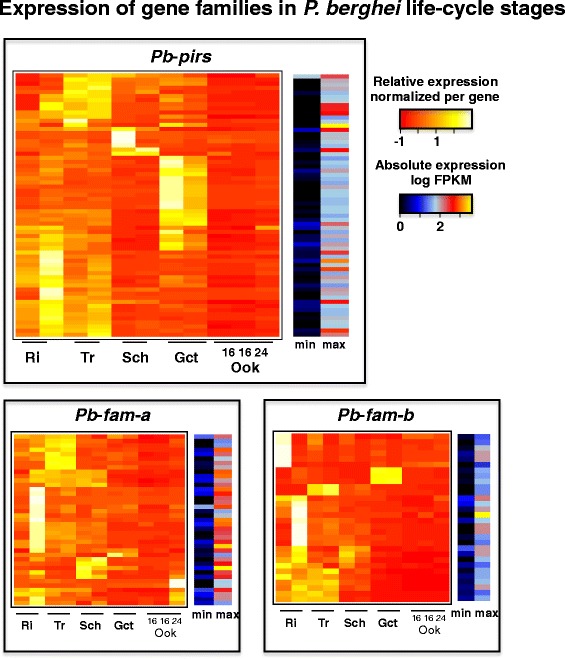
**Expression of members of three large RMP multigene families in different life cycle stages.** Temporal expression patterns of members of the three largest *Pb*A multigene familes (*pir*, *fam-a* and *fam-b*) in different life cycle stages as visualized by heat maps of RNA-seq data. The expression (FKPM) values of genes over the life-cycle stages (yellow-red) are normalised per gene. The min/max column values are the log minimal and log maximal FPKM values for each gene. Only genes with an FPKM above 21, in all conditions, were included. FKPM, fragments per kilo base of exon per million fragments mapped; RMP, rodent malaria parasites.

**Figure 4:**
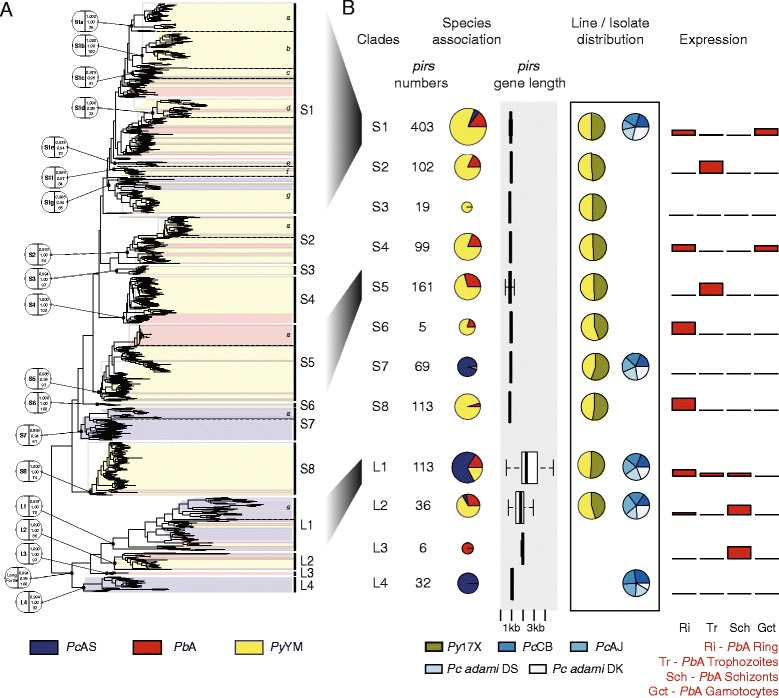
**Features of the RMP** ***pir*** **multigene family A) Phylogenetic tree of RMP** ***pir*** **s** ***,*** **showing the different clades (S1 to 8, L1 to 4) and separation of the ‘long’ (L) and ‘short’ form (S)** ***pir*** **s. B)** Features of the different RMP *pir* clades. For each clade we show the total number of RMP *pir*s followed by their distribution (pie charts) in the three species and the distribution of gene lengths (box plots). In addition, pie charts show the relative abundance of clades in the different isolates/lines of *P. chabaudi* and *P. yoelii*. The expression bar plots (red bars) visualise the expression of the *pir*s of different clades in the different life cycle stages (except for the ookinete stage since expression/FPKM values are below the cut of level of 21). A *pir* is assigned to a life cycle stage based on the highest FPKM value. The height of the expression bar represents the percentage of all *pir*s in that clade. FKPM, fragments per kilo base of exon per million fragments mapped; RMP, rodent malaria parasites.

We analysed the expression patterns of all members of the three largest multigene families, *fam-a, fam-b* and *pir* in the *Pb*A life cycle stages using heat maps of the RNA-seq data. This revealed distinct transcription patterns both between the gene families and also between members within a family (Figure 3). All three families show strongly reduced transcription in ookinetes. Whereas most *RMP-fam-a* and *RMP-fam-b* members had reduced transcript levels in gametocytes compared to asexual blood stages, a large cluster of *pir* genes were up-regulated (at least a fold change of 2) in gametocytes. Expression patterns are not only different between asexual and sexual stages but also between different asexual stages, for example distinct *pir* gene clusters are up-regulated in schizonts. Distinct transcription patterns in different life cycle stages of gene clusters may indicate functional differences between members of a single gene family. With the new genome assemblies we were able to determine the total number of *pir*s and their structure and spatial organization more precisely (Table 2; Additional file 4). In *Pc*AS and *Pb*A the total number of *pir*s (excluding pseudogenes) is 194 and 100, respectively, whereas in *Py*YM this gene family is greatly expanded to 583 copies. In Additional files 12, 13 and 14 the chromosomal distribution of all *pir*s is shown. Most *pir* genes share a similar structure across the different species with a short first exon, long second exon and a third exon encoding a trans-membrane domain. They lack a PEXEL-motif and all *pir*s are chromosomally arranged such that they are transcribed in a centromere to telomere direction except for several members of *Pc*AS (these are ‘long-form’ *pir*s of clade L1; see below). A number of *pir*s have long low complexity regions in the predicted extracellular domain and a few *Pc-pir*s have a four exon structure (see Additional file 3C). Remarkably, as stated earlier, the *Pb*-*pir* genes include a large number (88 genes; 44%) of pseudogenes and nearly half (35 genes; 18%) of these *Pb*-*pir*s are contained within the 2.3 kb subtelomeric repeat described above (Figure 1B). To analyse whether the differential expression of groups of *pir* members was associated with definable sequence differences (and possibly functional differences) between *pir*s we undertook a detailed phylogenetic analysis of all RMP *pir*s (including predicted pseudogenes). Estimations of Maximum Likelihood (ML) phylogeny based on nucleotide sequences or amino acid sequences and an estimation of a Bayesian phylogeny resulted in a phylogenetic tree with a robust separation of ‘long-form’ and ‘short form’ *pir*s (Figure 4; Additional file 15). We identified 12 clades in the phylogeny that have robust support; four long-form clades (L1 to 4) with a mean *pir* length ranging from 1,062 to 2,369 aa and eight short-form clades (S1 to 8) with a mean length ranging from 786 to 952 aa. Most long-form *pir*s have an extended repetitive region located within the second exon, downstream of the core *pir* domain and upstream of the transmembrane region. All RMP species have both short- and long-form *pir*s, indicating that the presence or absence of an extended repetitive region has evolved once and defines a principle division in *pir* diversity. Many clades are dominated by *pir*s from one species, particularly *Py*YM (for example, clades S1, S4, S8; Figure 4). Yet, even such clades contain rare sequence types from *Pb*A (for example, S1d, S2, S8) or *Pc*AS (S1g) indicating that these lineages originate from the RMP ancestor and probably expanded after speciation. Both *Pc*AS and *Pb*A appear to have experienced their own specific expansions after speciation (for example, S4, S5, S7). The maintenance of orthology within clades, in the presence of frequent gene conversion (see Discussion section), may indicate that selection pressure maintains structural differences between *pir*s, for example diversifying selection under immune pressure or purifying selection on functional diversity. The observation that the ratio of the different *pir* clades is highly similar in the five, highly diverse, isolates of the two *P. chabaudi* subspecies (Figure 4) supports the presence of selective pressures that maintain the clade structure of *pir* genes. We next analysed whether the *pir* expression patterns in *Pb*A blood stages were correlated with structural differences between *pir*s*,* by comparing the RNA-seq expression patterns with the phylogenetic clades. We observed that *pir*s that are predominantly expressed in gametocytes mainly belong to only two clades of the small-form *pir*s, S1 and S4, whereas genes up-regulated in schizonts are mainly long-forms of clades L1, L2 and L3 (see Additional file 16). The stage-specific up- or down-regulation of expression of clusters of structurally different *pir*s support the hypothesis of the existence of functional diversification within the *pir* family and is in agreement with other observations indicating that differences in *pir* sequences are associated with different functional properties [44,45].

## Discussion

By extensive re-sequencing and annotation we have generated three high quality RMP reference genomes with nearly all core genes as complete gene models and a much improved and almost complete representation of chromosomal subtelomeric regions. These reference genomes will greatly enhance the use of RMP as model organisms in malaria research. We provide full-length gene models for more than 98% of predicted protein-coding genes. The approximately 60% of genes with functional annotation is comparable to the percentage of functionally annotated genes in the *P. falciparum* 3D7 reference genome and a high percentage (approximately 90%) of the predicted RMP proteins have orthologs in primate malaria species. It is this high level of orthology between RMP and primate malaria genomes that strongly supports RMPs as models in experimental approaches to characterize the *Plasmodium* gene function. Similarly, the genome-wide RNA-seq data from different RMP developmental stages is a valuable resource to further analyse *Plasmodium* gene function and the regulatory networks underlying the multiple differentiation pathways of *Plasmodium*. The RNA-seq studies presented here provide information on gene expression at an unprecedented depth and breadth of coverage of multiple blood stages and ookinetes. Previously, only a few large-scale transcriptome (microarray) analyses of *P. berghei* blood stages and ookinetes had been performed [17,46]. These studies were based on a highly fragmented draft *P. berghei* genome and, therefore, expression data were only generated for about half of all *P. berghei* genes. In addition, important/valuable large scale transcriptome studies have been performed on RMP life-cycle stages, such as sporozoites and liver stages [47-49]. These life-cycle stages would also benefit from re-examination using the latest RMP genome assemblies we provide in this study.

Our studies reveal that large scale changes in gene expression occur in ookinetes between 16 and 24 hours after fertilization, possibly required for the differentiation of (retort-form) zygotes into the mature ookinetes. The strong up-regulation in mature ookinetes of transcripts involved in ribosome biogenesis and protein translation suggest that the mature ookinete generates transcripts for proteins required after the ookinete has traversed the mosquito midgut wall and starts its rapid transition into the oocysts, possibly using mechanisms of translational repression similar to those in gametocytes [50,51] and sporozoites [52,53]. What these three stages have in common is that they are fully differentiated cells that will undergo rapid cellular differentiation and/or growth expansion upon entering a new environment. Whether mature ookinetes store repressed transcripts requires further investigation.

The additional sequence data from multiple RMP isolates will help to further unravel gene function and establish relationships between phenotypic traits and genotypic diversity. The near absence of polymorphisms within the genomes of *P. berghei* isolates was unexpected. Low sequence diversity of a limited number of genes of *P. berghei* isolates had been reported previously and it was proposed that this may result from cross-contamination of *P. berghei* isolates in the laboratory after isolation [54,55]. However, this seems unlikely as one line would have needed to be mislabelled with the names of all other isolates, then all these mislabelled lines would have had to be sent to all the different laboratories worldwide replacing the ‘correct’ isolates that may have existed in their recipient laboratories. However, sequencing of additional stocks from these isolates, which were frozen in different laboratories soon after isolation from the natural host, may reveal whether low sequence diversity is due to cross-contamination. The *P. berghei* isolates we have sequenced were obtained from other laboratories (SP11, NK65) and they also show a similar lack of sequence polymorphism. In contrast, the isolates of *P. chabaudi* exhibit considerable genotypic diversity. These *P. chabaudi* isolates exhibit differences in virulence, RBC invasion, growth rates and immunogenic profiles [7,56-60] and further studies, for example using linkage or quantitative trait loci (QTL) analyses [33,61-63], will facilitate identification of genes associated with defined phenotypes. For RMP species there is evidence that differences in virulence are associated with differences in RBC invasion [2,7,56,60,64]. For example, *P. yoelii* virulence has been associated with mutations in proteins involved in RBC invasion [7,65,66]. Interestingly, we found extensive sequence polymorphism in *P. chabaudi* genes encoding such proteins. While much attention is given to the role of exported proteins of multigene families and virulence in both human and RMP, further analysis of RMP proteins that regulate invasion phenotypes may reveal novel mechanisms that underlie virulence.

The new sequence data allowed for a much improved annotation of chromosomal subtelomeric regions and to better define the different subtelomeric multigene families. In addition to the large *pir* gene family, all three RMP contain an expanded gene family encoding exported proteins, *fam-a*, with orthology to a single-copy gene in primate malarias, which contains a START-domain (steroidogenic acute regulatory-related lipid transfer domain; [67]). START-containing proteins of eukaryotes are involved in the transfer of phospholipids, ceramide or fatty acids between membranes [68]. A START domain has also recently been identified in an exported, PEXEL-containing, *P. falciparum* protein that was shown to transfer phospholipids [69]. The single-copy RMP orthologs of this gene (PF3D7_0104200) also contain a PEXEL-motif, indicating that phospholipid-transporting proteins are exported into the RBC in both primate malarias and RMP. *P. chabaudi* contains an additional, highly expanded, gene family that contains domains involved in phospholipid/fatty acid metabolism. These genes, encoding putative lysophospholipases, lack a PEXEL motif; however, for several *P. berghei* orthologs as well as lysophospholipases of *P. falciparum* there is evidence for their export into the host RBC [29,70]. Combined, these observations indicate the importance of phospholipid/fatty acid metabolism/transport mediated by *Plasmodium* proteins exported into the RBC cytosol. Why such genes have been differentially expanded into multigene families in different species remains to be investigated.

The *pir* family is the largest RMP multigene family and is shared with human and non-human primate species *P. vivax, P. knowlesi and P. cynomolgi* [20-23]. PIR proteins are exported into the RBC in the absence of a PEXEL-motif, and there is evidence that they are located on, or close to, the RBC surface or dispersed in the RBC cytoplasm [24-26,29,71,72]. The function of *pir*s is unknown and no functional domains have been identified so far. Recently, it has been shown that in *P. chabaudi* a change in virulence was associated with differential expression of members of the *pir* multi-gene family [27]. It has been suggested that PIRs are transported to the surface of infected RBC and play a role in RBC sequestration comparable to the role of the *Pfemp1* gene family of virulence factors in *P. falciparum*. However, for several *P. berghei* PIRs a direct role in RBC sequestration is unlikely since no evidence was found for their location on the RBC surface although they were exported into the RBC cytoplasm of both sequestering asexual blood stages and non-sequestering gametocytes [29]. For *P. vivax* PIRs it has been shown that different members have distinct subcellular locations in the infected RBC [26]. These observations indicate that functional differences may exist between members of the PIR family. Phylogenetic analyses support the possibility of functional differences between the PIRs. A recent phylogenetic analysis of the newly annotated *Pc*AS *pir*s identified two distinct *pir* sub-families (A and B), which contain distinct amino acid sequence motifs [44]. Our phylogenetic analyses included *pir*s from all three RMP species and resulted in the identification of a number of different clades. The presence of clearly distinguishable clades indicates that structural differentiation exists among *pir*s and that this evolved prior to the separation of the RMP species. Our observations of the stage-specific up- or down-regulation of expression of clusters of structurally different *pir*s in *different blood stages* supports the hypothesis that there is functional diversification within the *pir* family and that purifying selection plays a role in shaping this family. By including multiple species in the *pir* phylogeny it is clear that this gene family is subject to rapid turnover, that is, gene gain and loss, indicating the absence of strong selective forces that would result in distinct orthologous groups/clades that are shared and maintained in different species for functional reasons. Gain of *pir* genes in different species is evident in the multiple species-specific expansions of clades. Assuming that the common ancestor had a *pir* family equal in abundance and diversity, the relatively limited instances of orthology (12 clades) indicates significant losses of ancestral sequence types. A plausible explanation for both the abundance of species-specific sequences and the paucity of ancestral sequences is a continual process of gene turnover driven by gene conversion, a mechanism that has been proposed for *pir*s of *P. chabaudi* [44] and which was evident in each of the clades revealed in this study (data not shown). The effect of frequent gene conversion is the replacement of ancestral sequence types with species-specific sequences, which results in distinct species-specific clades without orthology. Loss of orthology is only resisted when selective forces maintain structurally distinct *pir*s, which we propose, explains the presence of the (limited) orthology between *pir* clades of the different RMP species. The improved annotation and phylogeny demonstrating clusters of structurally different *pir*s in all RMP combined with expression profiles are powerful data that can help to further delineate function, the relationship of expression with virulence and how the (species-specific) expansion of the *pir*s is related to distinct selective pressures.

## Conclusions

To maximise the utility of RMP we have greatly improved the genome assemblies of *P. berghei* and *P. chabaudi*, comprehensively sequenced the *P. yoelii* YM genome, sequenced multiple RMP isolates and generated in-depth expression data from multiple RMP life-cycle stages. Comparison of the RMP and *P. falciparum* genomes and RNA-seq mapping permitted gene annotation at base-pair resolution and has defined the level of orthology between RMP and human parasite genomes. The very high level orthology between RMP and human malarias (both in genome structure and gene content) supports the use of highly tractable RMPs as experimental models to characterize the function of the very many *Plasmodium* genes that remain uncharacterised.

Only a few large-scale transcriptome (microarray) analyses of different *P. berghei* life-cycle stages had previously been performed. Moreover, these studies were based on highly fragmented draft RMP genomes and consequently, for example, for one of the most well studied RMP, *P. berghei*, gene expression data was only mapped to about half of all *P. berghei* genes that have now been characterised. The RNA-seq studies we present provide information on gene expression, at an unprecedented depth and breadth of coverage, of multiple life cycle stages and provide the foundational data needed for the performance of large-scale analyses of gene regulatory networks that underlie cellular differentiation.

We show that extensive genotypic diversity exists between *P. chabaudi* isolates making this species an excellent organism to study genotype-phenotype relationships. Differences in virulence red blood cell (RBC) invasion, growth rates and immunogenic profiles exist between parasites of these isolates. Therefore, studies, such as linkage or quantitative trait loci analysis, are now possible to help identify genes associated with these defined phenotypes. For RMP species there is evidence that differences in virulence are associated with differences in RBC invasion, and, indeed, we find extensive sequence polymorphism in *P. chabaudi* genes encoding proteins involved in RBC invasion. Much attention is given to the role of exported proteins of multigene families and virulence in both human and RMP (for example, *var*, *pirs*), and analysis of differences between RMP proteins, that regulate invasion phenotypes, may reveal novel mechanisms that underlie virulence.

Full-length chromosomal annotation has permitted a comprehensive classification of all RMP subtelomeric multigene families. Our analyses indicate that most, if not all, RMP subtelomeric genes (apart from the RBP family) encode proteins exported out of the parasite; however, most lack a canonical PEXEL-motif. The presence of large numbers of PEXEL-negative exported proteins indicates alternative export mechanisms possibly common to all *Plasmodium* species. Investigations with highly tractable RMP species can therefore be used to understand these mechanisms better.

Our analyses of the phylogeny and expression of the largest RMP multi-gene family, the *pir*s, indicates functional diversification between members of the *pir* multigene family (this gene family is conserved between human/primate and RMP malaria species). Our new *pir* annotation and phylogeny demonstrates that clusters of structurally different *pir*s are differentially expressed. This is powerful data that can help to better understand their function, the relationship of *pir* expression with virulence and how the (species specific) *pir*s expansion is related to different selective pressures.

## Methods

### Animal experiments and parasites

All animal experiments performed in the Leiden malaria Research Group were approved by the Animal Experiments Committee of the Leiden University Medical Center (DEC 07171, DEC 10099). The Ethics Statement for *P. yoelii* YM and *P. yoelii* 17X: all animal work protocols were reviewed and approved by the Ethical Review Panel of the MRC National Institute for Medical Research and approved and licensed by the UK Home Office as governed by law under the Animals (Scientific Procedures) Act 1986 (Project license 80/1832, Malaria parasite- host interactions). Animals were handled in strict accordance with the ‘Code of Practice Part 1 for the housing and care of animals (21/03/05)’ available at [73]. The numbers of animals used was the minimum consistent with obtaining scientifically valid data. The experimental procedures were designed to minimize the extent and duration of any harm and included predefined clinical and parasitological endpoints to avoid unnecessary suffering. The study of *P. chabaudi* DNA and RNA was carried out in strict accordance with the UK Animals (Scientific Procedures) Act 1986 and was approved by the Ethical Committee of the MRC National Institute for Medical Research, and the British Home Office (PPL: 80/2538).

For sequencing of the RMP reference genomes the following were used: for *Pb*A the cloned reference line cl15cy1 of the ANKA isolate of *P. berghei* [11]; for *Pc*AS the 2722 clone of the AS isolate of *P. chabaudi chabaudi* (cloned after mosquito-transmission in 1978 and obtained from D. Walliker, University of Edinburgh, Edinburgh, UK); for *Py*YM the cloned 17XYM line of the YM line of *P. yoelii yoelii* [74]. In Additional files 6 and 7 details are provided of the other RMP isolates/lines that have been sequenced.

### Sequencing, assembly and annotation

Sequencing was performed using Sanger capillary, Illumina and 454 sequencing. Sequence assemblies were performed using different assemblers [75,76], which were improved automatically using a number of configuration tools [77-82] and manual inspection. First pass annotation was performed through a combination of *ab initio* gene finding via Augustus [83] and transfer of annotation through orthology using RATT [80]. Gene models of the three reference genomes were corrected manually using RNA-Seq and orthologous information. Details of the assemblies and annotation are provided in Additional file 6. To define the orthologous and paralogous relationships between the predicted RMP proteins and those of human/primate malaria species OrthoMCL [84] was used. The presence of a PEXEL-motif was determined using the updated HMM algorithm ExportPred v2.0 with a cutoff value of 1.5 [28]. Classification of the RMP multigene families was done through manual inspection of conserved domains (Interpro) and gene structure. SNPs in the genomes of these parasites were called by mapping the reads against their respective reference genomes, ignoring low complexity and repetitive regions. From the SNPs the Ka/Ks ratio was calculated for the *P. chabaudi* isolates with the Bio::Align::DNAStatistics Perl module.

### Transcriptomics

RNA was collected from multiple synchronized blood stages [85] and purified gametocytes and ookinetes [86] of *Pb*A, from *Pc*AS blood stages (late trophozoites), isolated from different mice as described [44] and from *Py*YM late blood stages of two parasite lines (the cloned YM line and mutant PY01365-KO) [8]. RNA was sequenced as described [8,44,87,88]. To correct gene models and to compare the expression between samples, each sample was first mapped against its reference genome using TopHat [89] (version v2.0.6, parameter -g ). For the resulting output a custom Perl script was written to detect errors in the annotation and to find new or alternative splice sites. To determine transcript abundance FPKM values were calculated for all genes (FPKM: fragments per kilo base of exon per million fragments mapped) using Cufflinks [90]. Accepting 10% of the intron as real signal, a cut-off FPKM value of 21 over all RNA-seq samples was determined. See also Additional file 6 for a detailed description of the generation and analysis of the RNA-seq data.

Heatmaps were generated with FPKM values of each gene and condition, using the heatmap.2 function of the gplots package. Correlation plots were done in R (Foundation for Statistical Computing; [91]) and generated with the corrplot function of the corrplot R library. Only genes were included that had one to one orthology in the three rodent species. For differential expression we used cuffdiff [90] (v2.0.2, with parameters -u -q) to compensate for GC variation and repetitive regions. GO enrichment of differentially expressed genes was performed in R, using TopGO. As a GO-database the predicted GO-terms from the reference RMP genomes were used.

### Phylogenetic analyses of *pir*s

All full-length RMP *pir* coding sequences, including predicted pseudogenes, were used. Translated nucleotide sequences for 1,160 genes were aligned in ClustalW [92]; all multiple alignments were manually edited to resolve all frame-shifts. Non-homologous positions at the N-terminus were removed by curtailing the alignment to the N-terminal-most conserved cysteine position. Non-homologous repetitive motifs were removed from ‘long-form’ PIRs (that is, 188 proteins >1,200 amino acids in length). The resultant 1,266-character alignment constitutes the conserved core of all PIRs and almost the complete repertoire of ‘short-forms’ (that is, <1,200 amino acids in length and 972/1,160 genes). A Maximum Likelihood phylogeny was estimated from the nucleotide sequence alignment using RAxML v7.0.4 [93] using a GTR + G model. Node support was assessed using 100 non-parametric bootstrap replicates [94]. A Bayesian phylogeny was estimated using MrBayes v3.2.1 [95] with a GTR + G model for a subsample of *pir* nucleotide sequences (MCMC settings: Nruns = 4, Ngen = 1,000,000, sample burnin = 1,000, and default prior distribution). See also Additional file 6 for a detailed description of the phylogenetic analyses.

### Accession numbers

All the raw data used in the assemblies of the genome and the RNA-seq data have been deposited with accession numbers shown in Additional file 17. The reference genomes have the following accession numbers: *P. chabaudi* chromosomes: LK022878-LK022893 and scaffolds: LK022855-LK022877, *P. berghei* chromosomes: LK023116-LK023131 and scaffolds: LK022894-LK022977; *P. yoelii* YM chromosomes: LK934629-LK934644 and scaffolds: LK023132-LK023312 and *P. yoelii* 17X chromosomes: LM993655-LM993670 and scaffolds: LK022978-LK023115.

## Additional files

Additional file 1:
**Novel putative protein coding genes which were absent in the previous genome annotations of** ***P. berghei*** **ANKA and** ***P. c. chabaudi*** **AS.**
Additional file 2:
**RMP genes with a PEXEL motif as identified by using the updated HMM algorithm ExportPred v2.0.**
Additional file 3:
**A. Chromosomal organization of the expanded** ***fam-d*** **multigene family in the internal region of chromosome 9.** The *Pc*AS, *Py*YM and *Pb*A genomes contain 21, 5 and 1 copy (PBANKA_091920), respectively (shown in blue). **B**. An ACT (Artemis Comparison Tool) comparison of syntenic centromeric regions (green) of chromosome 7 of *Pb*A and *Pc*AS and chromosome 6 of *P. falciparum* 3D7, showing size, location and GC-content. Grey bars: forward/reverse DNA strands. The red lines represent sequence similarity (tBLASTx) (related to Table 1). **C**. Structural organization of several types of full-length *pir* genes in *Pb*A (*birs*), *Pc*AS (*cirs*) and *Py*YM (*yir*s). Exons: yellow boxes; with introns: linking lines. The IDs shown represent a single example. Additional file 4:
**The different (subtelomeric) multigene families in the genomes of** ***P. berghei*** **ANKA,** ***P. c. chabaudi*** **AS and** ***P. y. yoelii*** **YM:** ***pir; fam-a; fam-b; fam-c; fam-d; ETRAMP; reticulocyte binding protein, putative; lysophospholipase; PCEMA1; haloacid dehalogenase-like hydrolase; ‘other subtelomeric genes’.***
Additional file 5:
**Putative orthologs (OrthoMCL) of RMP and primate malaria proteins: 1) RMP proteins shared with at least one primate malaria protein; 2) RMP specific proteins (no orthologs in primate malarias); 3) primate malaria specific proteins (no orthologs in RMP).**
Additional file 6:
**Additional methods.**
Additional file 7:
**Information on RMP isolates/lines used in the study for genome sequencing and RNAseq analyses (see also Additional file** 6 **).**
Additional file 8:
**Genes with single nucleotide polymorphisms (SNPs) in: 1) different stabilates of** ***P. berghei*** **isolates/lines (NK65NY, NK65 E, SP11 A, SP11 RLL A, K173; compared to the genome of** ***P. berghei*** **ANKA); 2) in** ***P. y. yoelii*** **17X (compared to the genome of** ***P. y. yoelii*** **YM); and 3) in different isolates of** ***P. c. chabaudi*** **(AJ, CB) and** ***P. c. adami*** **(DS, DK) (compared to the genome of** ***P. c. chabaudi*** **AS).**
Additional file 9:
**RNA-seq analysis: mRNA abundance (FPKM values) of all genes in the different life cycle stages of** ***P. berghei*** **ANKA,** ***P. c. chabaudi*** **AS and** ***P. y. yoelii*** **YM.**
Additional file 10:
**Differentially expressed genes in 16 hour ookinetes compared to 24 hour ookinetes of** ***P. berghei*** **ANKA and GO-annotation of up- and down-regulated genes.**
Additional file 11:
**Overview and details of spliced transcripts and alternative splicing (AS) in the RMP genomes based on RNA-seq analyses.**
Additional file 12:
**Distribution of members of different multigene families on chromosomes of** ***Pb*** **A.**
Additional file 13:
**Distribution of members of different multigene families on chromosomes of** ***Pc*** **AS.**
Additional file 14:
**Distribution of members of different multigene families on chromosomes of** ***Py*** **YM.**
Additional file 15:
**RMP** ***pir*** **s in different phylogenetic clades.** Classification of *pir* genes by clade and by species, with information on predicted protein sequence length, base composition and codon usage. Additional file 16:
**Expression of PIRs in relation to their phylogenetic relationship.** Heat maps of expression (FPKM values >21; normalized by gene) of all *Pb*A *pir*s in different life cycle stages in association with their location in different clades (L, S) of the phylogenetic tree (black boxes). Ri: ring; Tr: Trophozoite; Sch: Schizont; Gct: Gametocyte; Ook: Ookinetes (16 and 24 hour ookinetes). Additional file 17:
**Description of genomic and RNA-seq reads and their Accession numbers.**
