# Supplementary material for: A comprehensive evaluation of rodent malaria parasite genomes and gene expression
Source: BMC Biol. 2014 Oct 30;12:86. doi: 10.1186/s12915-014-0086-0 (PMC4242472; doi:10.1186/s12915-014-0086-0)
Supplement: Additional file 3: — A. Chromosomal organization of the expanded fam-d multigene family in the internal region of chromosome 9. The PcAS, PyYM and PbA genomes contain 21, 5 and 1 copy (PBANKA_091920), respectively (shown in blue). B. An ACT (Artemis Comparison Tool) comparison of syntenic centromeric regions (green) of chromosome 7 of PbA and PcAS and chromosome 6 of P. falciparum 3D7, showing size, location and GC-content. Grey bars: forward/reverse DNA strands. The red lines represent sequence similarity (tBLASTx) (related to Table 1). C. Structural organization of several types of full-length pir genes in PbA (birs), PcAS (cirs) and PyYM (yirs). Exons: yellow boxes; with introns: linking lines. The IDs shown represent a single example. [file 12915_2014_86_MOESM3_ESM.pdf]

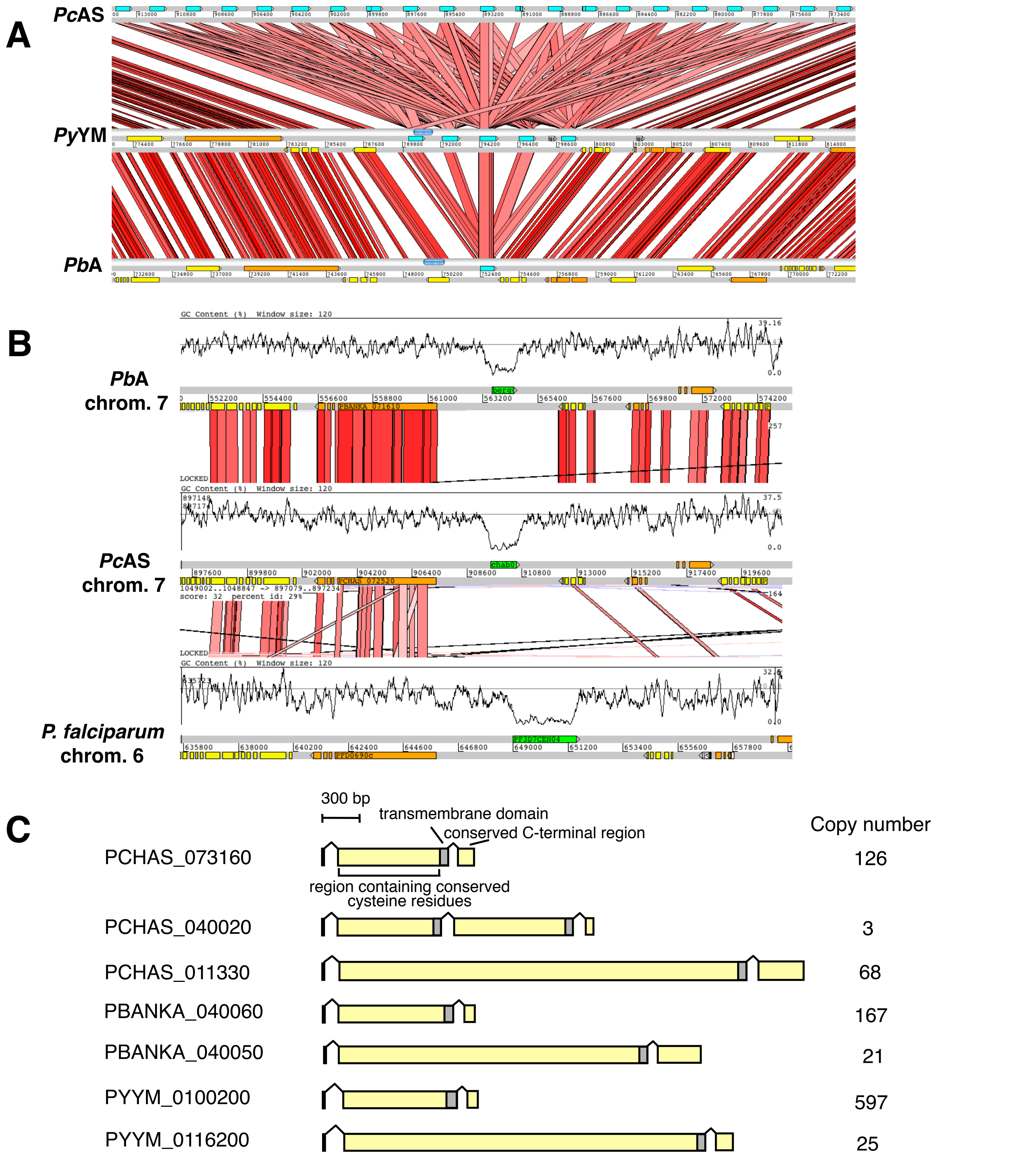

Figure S1

A. Chromosomal organization of the expanded *fam-d* multigene family in the internal region of chromosome 9. The *Pc*AS, *Py*YM and *Pb*A genomes contain 21, 5 and 1 copy (PBANKA_091920), respectively (shown in blue) (related to Table 2)

B. An ACT (Artemis Comparison Tool) comparison of syntenic centromeric regions (green) of chromosome 7 of *Pb*A and *Pc*AS and chromosome 6 of *P. falciparum* 3D7, showing size, location and GC-content. Grey bars: forward/ reverse DNA strands. The red lines represent sequence similarity (tBLASTx)(related to Table 1).

C. Structural organization of several types of full-length *pir* genes in *Pb*A (*birs*), *Pc*AS (*cirs*) and *Py*YM (*yir*s). Exons: yellow boxes; with introns: linking lines. The IDs shown represent a single example.
