# Supplementary material for: A comprehensive evaluation of rodent malaria parasite genomes and gene expression
Source: BMC Biol. 2014 Oct 30;12:86. doi: 10.1186/s12915-014-0086-0 (PMC4242472; doi:10.1186/s12915-014-0086-0)
Supplement: Additional file 6: — Additional methods. [file 12915_2014_86_MOESM6_ESM.docx]

**Additional file 6: Methods**

**Table of contents** **Genomics**

*Parasites for the RMP reference genomes*

*Additional RMP isolates/lines*

Preparation and s*equencing of DNA*

*Genome assembly of the RMP reference parasites*

*Genome annotation of the RMP reference parasites*

*Genome assembly of RMP isolates/lines*

*Genome annotation of RMP isolates/lines*

*Ka/Ks analysis*

**Transcriptomics**

*RNA preparation and sequencing*

*RNA-seq analysis*

**Analysis of genes, classification of multigene families and phylogenetic analyses** *Orthologous genes
 PEXEL*-motif
 *Classification of the multigene families*
 *Phylogenetic analyses*

**Genomics**

***Parasites for the RMP reference genomes***

For sequencing of the RMP reference genomes the following were used: for *Pb*A the cloned reference line cl15cy1 of the ANKA isolate of *P. berghei* [1]; for *Pc*AS the 2722 clone of the AS isolate of *P. chabaudi chabaudi* (cloned after mosquito-transmission in 1978 and obtained from D. Walliker, University of Edinburgh, Edinburgh, UK); for *Py* YM the cloned YM line of *P. yoelii yoelii*; this is a highly virulent line derived from the *P. y. yoelii* 17X isolate and cloned in Edinburgh [2].

***Additional RMP isolates/lines***

Genome sequences were generated from a number of additional isolates/lines of *P. berghei* (NK65 NY, NK65 E, K173, SP11, SP11 RLL), *P.* *chabaudi* *chabaudi* (AS, AJ, CB) and *P.* *chabaudi adami* (DK, DS). In addition we generated additional sequence data for *P. y. yoelii* 17X. Please see **Additional file 7** for isolate information including the origin, dates of exchange of lines between labs and the laboratory where the DNA/RNA was prepared. Further details of the original RMP isolates are described in Beale *et al*. [3], Killick-Kendrick [4] and Ramiro *et al*. [5]. For most of the original *P. berghei* isolates no details are available of the exact history of the maintenance of these lines in different laboratories. Stabilates of the *P. berghei* isolates KSP11, LUKA, SP11 and K173 from the University of Edinburg collection [5] do not produce gametocytes (unpublished observations, C.J.J) and were not included for sequencing. Of the *P. c. chabaudi* isolates AS is the mildest, AJ is more virulent and CB is the most virulent, where ‘virulence/mildness’ refers to growth rate of blood stage parasites and their pathogenicity/lethality [6]. The line of the *P. c. adami* DS isolate is virulent (lethal at day 10 by blood infection; [7]).
Genomic DNA was purified from (mixed) blood stages from the five following *P. berghei* stabilates:
**1.** The K173*cl1* line of the *P. berghei* K173 isolate. This laboratory line, obtained from Nijmegen (The Netherlands) does not produce gametocytes and schizonts do not show a CD36-mediated sequestration phenotype like ANKA schizonts [8]. The karyotype of the Nijmegen K173 parasites has been analysed and is distinct from that of ANKA and NK65 parasites [9]. We were unable to obtain a stabilate of the original K173 isolate that produces gametocytes. Different K173 lines from the University of Edinburg collection show comparable karyotypes to the Nijmegen K173 line (unpublished observations C.J.J).
**2.** The NK65 NY line of the *P. berghei* NK65 isolate. Different laboratories use NK65 lines that originate from NK65 parasites maintained and propagated in New York [10]. This NK65 NY (‘New York’) line produces gametocytes and does not cause experimental cerebral malaria (ECM) in ECM-sensitive C57Bl6 mice. The NK65 NY line used for genome sequencing was provided by Robert Menard (Institute Pasteur, Paris) in 2011 to the Sanger Institute.
**3.** The NK65 E line of the *P. berghei* NK65 isolate. The NK65 E parasites used for genome sequencing had been obtained by Philippe van den Steen, (Leuven, Belgium) from the University Edinburgh collection [5] and sent to Leiden (The Netherlands) in 2011. This line produces gametocytes and does not cause ECM in C57Bl6 mice [11]. These parasites were used in Leiden to generate a cloned transgenic line that contains the *hdhfr-tgdhfr* selection cassette integrated into the silent *230p* gene locus (PBANKA_030600). This so-called NK65 GIMO-motherline (1995cl1) has been used for sequencing.
**4.** The SP11 cl1 A line of the *P. berghei* SP11 isolate. A stabilate of the SP11 isolate was obtained from the Institute of Tropical Medicine in Antwerp (Belgium). This stabilate was sent to Leiden in 2011 and cloned in Leiden (SP11 clone 1 Antwerp). This cloned line (SP11 cl1 A) produces gametocytes and schizonts that show a CD36-mediated sequestration phenotype (unpublished observations C.J.J) like *P. berghei* ANKA schizonts [12]. The cloned line has been used for sequencing.
**5.** The SP11-RLL A line of the *P. berghei* SP11 isolate. SP11-RLL is a pyrimethamine-resistant laboratory line of the SP11 isolate. A stabilate of SP11-RLL A (‘Antwerp’) was obtained from the Institute of Tropical Medicine in Antwerp (Belgium). This stabilate was sent to Leiden in 2011. Uncloned parasites of the SP11-RLL A line were used for sequencing. This line produces gametocytes.
For *P. chabaudi* the genomes of the following four isolates/lines from *P. c. chabaudi* and *P. c. adami* were sequenced (see the European Malaria Reagents Repository; [www.malariaresearch.eu](http://www.malariaresearch.eu) from where also live parasite stabilates are available. The information below is taken from this webpage). Genomic DNA was purified from (mixed) blood stages from the four following *P. chabaudi* isolates using a phenol/chloroform extraction protocol. They were also treated for RNase and stored in TE buffer pH8. White cells were depleted by passing the infected blood twice through Plasmodipur filters (EuroProxima, NL).
**1.** The AJ isolate of *P. c. chabaudi.* A cloned line (96AJ15) of the 166BY isolate. It was isolated from a wild caught *Thamnomys rutilans* captured near Bangui (Central African Republic (CAR), it arrived in Paris 15/3/69 and four days later at Edinburgh and stored frozen. It was passaged four times in *G. surdaster* and transmitted three times in *An stephensi*, and finally passaged 8x in mice. Cloning 14.5.73 (D. Walliker); 96AJ15 (frozen stabilate 1.6.73). See also http://www.malariaresearch.eu/reagents/rodent-malaria-line/plasmodium-chabaudi-chabaudi-aj-96aj15 for further information.
**2.** The CB isolate of *P. c. chabaudi* [5]. The cloned line 50CB2 of the CB isolate*.* It was isolated from a wild caught *Thamnomys rutilans* captured near Bangui and arrived Edinburgh 25/9/70. It was passaged into *G. surdaster*; frozen isolates, 9/10/70. 5x mouse passage, *An. stephensi* transmission; frozen stabilate,14/12/72. See also http://www.malariaresearch.eu/reagents/rodent-malaria-isolate/cb-isolate
**3.** The DK isolate of *P. c. adami*. The cloned line 19DK1 of the 556KA isolate (DK). Isolated in Congo-Brazzaville from *Thamnomys rutilans* 556KA. 2 mouse passages, deep-freeze, 4 mouse passages. Mice sent to Edinburgh 04.12.71; 1DK. -> 3x mouse passage*, An. stephensi* transmission; frozen stabilates, 20/11/73. Cloning 28/5/74; frozen stabilates 19DK1 and 19DK23, 10/6/74. See also http://www.malariaresearch.eu/reagents/rodent-malaria-line/plasmodium-chabaudi-adami-dk-clone
**4.** The DS isolate of *P. c. adami.* The cloned line 15DS12 of the 408XZ isolate (DS). It was isolated in Congo-Brazzaville, 28.10.72 from *Thamnomys rutilans*. Frozen blood sent to LSHTM by Irene Landau. Inoculated into mice in 28.9.73, sent to Edinburgh 18.10.73 (mouse 1DS). Cloned, 19/7/74; stabilates 15DS12. 10x mouse passage, 2x mosquito transmission; stabilates made on 2/7/82. Blood isolates of naturally-infected Thicket Rats captured in the CAR in 1965 (Landau & Chabaud). See also http://www.malariaresearch.eu/reagents/rodent-malaria-line/plasmodium-chabaudi-adami-ds-clone
*P. yoelii yoelii* originated from a single uncloned avirulent parasite isolated in 1965 in the Central African Republic and designated 17X. Between 1967 and 1975 in Edinburgh, two fast growth virulent lines of the parasite arose independently from this isolate and were characterized by their ability to develop in mature erythrocytes. The parasites were cloned.

1 *P. yoelii yoelii YM* a line cloned from 17XYM, genotype 1 (University of Edinburgh 1972-1973)

2. *P. yoelii yoelii* 17X, a line cloned from the 17X isolate (transferred from University of Edinburgh to NIMR, 1978). The isolate is slow growth and avirulent.

***Preparation and sequencing of DNA*1)** *Pb*A genomic DNA (gDNA) was collected from cultured mature schizonts, collected and purified as described [13]. In brief, infected blood with a parasitemia of 1-3% was collected by heart puncture from Wistar rats, leucocytes were removed with Plasmodipur filters, and cultured overnight at 37°C. Schizont-infected red blood cells (RBC) were separated from uninfected rbc using Nycodenz-gradient centrifugation. Approximately 1x10^9^ schizonts were resuspended in complete culture medium and passed through two CS columns (Miltenyi Biotec GmbH) of a VariaMACS magnetic cell separator for collecting schizonts as described [14]. The magnetic separation step was included to reduce leucocyte derived host-DNA contamination.
**2)** *Pc*AS genomic DNA (gDNA) was extracted from trophozoite-stage parasites. Infected blood with a parasitemia of 15-25% was collected from C57BL/6 mice as described [15]. Leucocytes were removed by passage through Plasmodipur filters and red blood cells removed by saponin lysis (0.15% saponin in PBS for 6 minutes on ice). RNA was removed by digestion with 100μg/ml RNase A in 50mM Tris pH7.5, 50mM EDTA pH8, 100mM NaCl, 0.5% SDS, 37°C, 30 minutes, and gDNA extracted by addition of 100μg/ml proteinase K, 45°C overnight, followed by phenol chloroform extraction and ethanol precipitation.
**3)** *Py*YM and *Py*17X gDNA were isolated from leukocyte-depleted, magnet-purified late stage parasite-infected erythrocytes. Briefly, infected erythrocytes were harvested at 15 to 25% parasitemia and passed through two Plasmodipur filters to remove leukocytes. Late stage parasitized erythrocytes were then purified using a MACS type-D depletion column and a SuperMACS II magnetic separator (Miltenyi Biotec GmbH). Infected erythrocytes were lysed in a buffer containing SDS and the DNA was purified by phenol chloroform extraction and precipitated with ethanol.
All the libraries sequenced are reported in **Additional file 17**. The table includes further information for each library, such as accession number, expected coverage and fragment size.

*Sanger sequencing*

Sanger capillary sequencing data were initially generated to produce the reference genomes *P. berghei* ANKA and *P. c. chabaudi* AS. The genomes were sequenced to approximately up to 12-fold coverage from pUC19 (with insert size 1-4 kb) and pMAQ1b_SmaI (with insert size 4-6 kb) genomic shotgun libraries using big-dye terminator chemistry on ABI3730 automated sequencers. End sequences from large insert fosmid libraries in pCC1Fos (insert size 38-42 kb) were used as a scaffold. Gap closure was performed for *P. berghei* ANKA using polymerase chain reaction (PCR). *P. c. chabaudi* AS was manually improved to ‘Noncontiguous Finished’ standard [16].

*Illumina sequencing*

Illumina libraries were generated using the amplification free protocol [17]. Libraries were sequenced on the Illumina Genome Analyser IIX for 76 paired end cycles or the Illumina HiSeq 2000 for 75 or 100 paired-end cycles using V4 or V5 SBS sequencing kit and proprietary reagents according to manufacturer's recommended protocol (<https://icom.illumina.com/>). Data were analysed from the Illumina sequencing machines using RTA1.6, RTA1.8 or GA v0.3 analysis pipelines.

*454 sequencing*

Paired-end (3 kb, 8 kb and 20kb) and shotgun 454 libraries were generated using standard Roche protocols ([www.454.com](http://www.454.com)) and sequenced using the 454 Life Sciences GS-FLX sequencer (Roche) except for the 20kb 454 library of *P. berghei* ANKA which was prepared and sequenced by Roche (Branford, CT, USA).

***Genome assembly of the RMP reference parasites***

All used data (including the accession numbers) are shown in **Additional file 17**.
**1.** *P. c. chabaudi* AS (*Pc*AS). Sequence assembly was performed using 8x Sanger data, using the Phusion assembler [18]. Following manual improvement a further 52 out of 69 gaps were closed using a combination of oligo-walking and PCR. Base correction was also manually performed followed by ICORN (Iterative Correction Of Reference Nucleotides [19]), which was run to correct single base and short indel errors.
**2.** *P. berghei* ANKA (*Pb*A). Sanger, 454 and Illumina sequencing data were assembled independently using Phusion, Newbler and Velvet [20] (parameter: k-mer of 55; -exp_cov auto; -cov_cutoff 8), respectively. Contigs were ordered against the new *Pc*AS assembly using ABACAS (Algorithm-Based Automatic Contiguation of Assembled Sequences [21]. ABACAS merged contigs of the three different technologies if two contigs overlapped by more than 500bp and with an identity > 99%. Sequence gaps were then closed automatically with IMAGE (Iterative Mapping and Assembly for Gap Elimination [22] and the Illumina reads. The genome assembly was further improved by manual finishing, which included i) correction of the synteny break between *Pb*A and *Pc*AS; and ii) manual scaffolding of the subtelomeric contigs using the 20kb 454 read pair data. Following manual improvement a further 67 out of 124 sequencing gaps were closed using a combination of oligo-walking and PCR. Base errors were corrected with ICORN.
**3.** *P. yoelii* YM: The Illumina reads of *Py*YM were assembled with Velvet (version 1.1.05, parameters: k-mer of 65; -min_pair_count 20 -ins_length 475 -exp_cov auto -cov_cutoff 10). Onto this new assembly, the reads were mapped (SMALT: version 0.5.5. parameters: k=13, s=2, -r 0 -x -y 0.8 -j 200 -i 800) and Velvet Columbus ran with the same parameters as the standard Velvet run. The resulting scaffolds were further scaffolded with SSPACE [23] using first the Illumina library (insert=475, s.d.=0.3. –n 31 –x 0, run iteratively with a decreasing k-mer for joining scaffolds, -k=20,10,10,7,7,5,5,5) and with a 454 3kb library (parameters as before, with the exception of a fragment size of 2700 bp and standard deviation of 0.6). Next sequencing gaps were closed (IMAGE) and base errors corrected (ICORN). After manual inspection of the assembly, the scaffolds were ordered with ABACAS against the *Pb*A reference genome.
***Genome annotation of the RMP reference parasites***Annotation was performed using Artemis and ACT software [24]. The *Pc*AS genome was manually annotated based on synteny with the *P. falciparum* 3D7 genome. For *Pb*A*,* gene models were transferred using RATT (Rapid Annotation Transfer Tool) [25]. The *Py*YM was annotated with RATT and with Augustus trained on a set of 250 manually curated genes. Functional assignments were extracted from literature or based on assessment of BLAST and FASTA similarity searches against public databases and searches in protein domain databases included in InterPro [26]. In addition, SignalPv3.0, TMHMMv2.0 and tRNA-scan were used to identify signal peptides, transmembrane domains and tRNA genes. To validate intron-exon boundaries a bespoke Perl script compared the evidence of RNA-seq data with the splice boundaries and differences between the script and the annotation were investigated manually. As further evidence the splice form of homologous genes in other *Plasmodium* species was considered. All Gene IDs from the previously published *Pb*A and *Pc*AS assemblies [27] were mapped with reciprocal blast to the new assemblies. Genes were assigned to the new gene models if the match of the former gene model was at least 20% of the length of the new gene with at least 70% identity. To define the orthologous and paralogous relationships between the predicted proteins of the three RMP reference genomes and of *P. falciparum*, *P. knowlesi* and *P. vivax*, the OrthoMCL protein clustering algorithm [28] was used.
***Genome assembly of RMP isolates/lines***
The genome of *P. y. yoelii* 17X was assembled as described above for the *Py*YM reference genome. For most of the analysis a subset of Illumina reads was used, representing 300x coverage. If not stated differently, we used a fragment size of 525bp (0.3 standard deviation) for the Illumina library. After the initial Velvet step (version 1.1.06, parameters: k-mer 91, -cov_cutoff auto -ins_length 520 -exp_cov auto -min_pair_count 10), the scaffolds were first orientated with the Illimina reads (parameters: Insert=515, s.d.=0.3. –n 31 –x 0 -k=60,30,20,10,10,7,7,5,5,5), then with the 8kb 454 library (Insert=7500, the other settings were the same as for Illumina) followed by ordering against the *Py*YM genome using ABACAS. To close sequencing gaps, first GapFiller ([29], parameters: 545bp fragment size and 0.3 deviation) was run, then IMAGE (Parameter, k-mer of 91 and 81, were run for 5 iterations, respectively; then k-mers of 71,61,51 and 41 were run for 3 iterations respectively) and finally PBjelly were run using the 454 reads. At this stage the assembly was evaluated automatically using REAPR (Recognising Errors in Assemblies using Paired Reads [30]), which breaks the assembly automatically if reads pairs are not distributed as expected. Scaffolds smaller than 500 bp were excluded. To obtain the final genome sequence, SSPACE, IMAGE, ICORN and REAPR were run again. To double check for possible missed sequences, reads were mapped against the final genome sequence and all mate pairs that did not map were assembled separately (bin assembly). Those contigs were joined into the assembly by scaffolding with SSPACE using the 454 8kb library. Possible mouse contamination was deleted by similarity comparison of scaffolds smaller than 20kb against the mouse reference genome (GRCm38), using SMALT (parameters: k=13, s=3, SMALT map –y 0.9 –r 1). During the assembly we observed errors in the mitochondrial and apicoplast genomes, as well as in the unordered contigs (bin pseudo sequence), which were corrected manually. Further, many frame-shifts in genes due to homopolymer tracks longer then 20bp were not corrected by ICORN. Those were also manually fixed and reported in the annotation.
*P. berghei* and *P. chabaudi* isolates: For the genomes of these isolates/lines we generated a completely automated pipeline. First, low quality regions for the reads were clipped with SGA version 0.9.1 ([31]; parameters: -m 51 --permute-ambiguous -f 3 -q 3). Those reads were assembled with Velvet iterating through the following k-mers: 85, 81, 71 and 55. The other parameters were: -exp_cov auto -ins_length 450 -ins_length_sd 30 -cov_cutoff 5 -min_contig_lgth 200 -min_pair_count 10. For the *P. berghei* isolates/lines a k-mer of 81 was used. For the *P. chabaudi* isolates we chose follow k-mer settings: AJ and DK 55, CB 61 and DS 65. To improve the assembly several tools were used, as described in PAGIT (Post Assembly Genome Improvement Toolkit [32]. First two rounds of iCORN2 [19] corrected single base pair errors and small indels. The resulting contigs were scaffolded with the reads using SSPACE [23] (parameters: as above using an iterative approach). Assembly errors were detected with REAPR [30], breaking the contig at each Fragment Coverge Distribution error (Parameter -l to also break contig errors). Those corrected contigs were ordered with ABACAS [21] against the above described reference genomes. To minimize false attribution of contigs in the subtelomeric regions, the subtelomeric sequence was replaced with Ns in the reference genomes. Next sequencing gaps were closed by twice seven iterations of GapFiller ([29], parameter -i 31) and six iterations with IMAGE, with two iterations of decreasing k-mers of 71, 55 and 41.

***Genome annotation of RMP isolates/lines***

The genomes of the RMP isolates/lines were annotated by transferring the annotation from their reference genomes (see above) using RATT (parameter “Species” and “Eukaryotic“configuration file). Although RATT has a correction model, some transferred gene models could not be corrected. Those gene models were excluded from the gene set. The gene finder software Augustus [33] was trained with the complete gene set of each reference genome to call gene models *ab initio*. Those models were compared with BLAST against the proteome of the RMP reference genome (annotation as described above) and the first BLAST hit was taken as annotation of function. Next the gene models of RATT and Augustus were merged. The RATT model was always chosen when two genes overlapped between both callers. Last, all chromosomes and contigs were joined into one sequence (union file) and provided an identifier for each gene.

The genomes can be found on the following ftp-site: <ftp://ngs.sanger.ac.uk/scratch/project/pathogens/Plasmodium/RMP/>

***Ka/Ks analysis***

Genomic reads were mapped with SMALT. Reads were realigned using GATK [34] and SNPs called with mpileup of the Samtools package [35], parameter: -Q 20 -d 2000 and varFilter -d 10 -D 2000). We further filtered SNPs to require confirmation from at least 10 reads with bases of high quality (Q>= 20) and ignored heterozygous sites where the allele frequency was below 0.8. SNP calls were ignored in repetitive regions (defined by a 70 bp word length) and in low complexity (Dustmaker [36]). The Ka/Ks (or dN/dS) ratio were calculated with the Bio::Align::DNAStatistics Perl module.

Files (in the variant calling format, VCF) containing the high quality variants can be found at: <ftp://ngs.sanger.ac.uk/scratch/project/pathogens/Plasmodium/RMP/>.

**Transcriptomics**

***RNA preparation and sequencing***

For RNA-seq analyses the following parasite lines were used:

1. For *Pb*A the cloned reference lines cl15cy1 [1], 2.34 [37] and 1037cl1 [38]. The latter line is a transgene reference parasite that contains the fusion gene *gfp-luciferase* in the silent *230p* locus and is made in the cl15cy1 background (see RMgm-32, [www.pberghei.eu](http://www.pberghei.eu)).

2. For *Pc*AS a cloned line of *P. c. chabaudi* (AS) [39].

3. For *Py*YM the cloned YM line and the mutant PY01365-KO line [40].

*Pb*A blood stages were obtained from synchronized *in vitro* cultures during the 22-24 hour asexual cycle [13]. Synchronized cultures were generated from the lines cl15cy1 and 1037cl1 (R = repeat samples). In brief, infected heart blood with synchronized ring forms (0-4h after invasion) was collected from Wistar rats as described [13], leucocytes were removed, and the blood cultured at 37°C for a period of 22 hours. Infected red blood cells were collected at the following time points: 4h, 16h, and 22h (in total 1-3 x 10^8^ parasites per sample). These time points correspond to ring forms (4h), mature trophozoites (16h; no nuclear division) and maturing schizonts and gametocytes (22h). In these cultures >85% of the rings develop into schizonts and 5-10% develop into gametocytes. In the samples prepared for RNA-seq 8% of the cl15cy1 parasites and 10% of 1037cl1 parasites developed into gametocytes as determined by microscopy with Giemsa-stained slides of the cultures at 26h. At 16h, no distinction can be made between asexual and sexual trophozoites based on their morphology (light/electron microscopy). The 22h sample consisted of dividing schizonts (2-16 nuclei) and ‘immature’ gametocytes [13]. The 4h sample consisted of >95% ring forms with <5% mature gametocytes (cl15cy1: 3%; 1037cl1: 4%; as determined by Giemsa-stained slides of samples at 4h). These mature gametocytes are ‘carried over’ in schizont cultures which had been used to establish the synchronized infections. In all experiments duplicate samples (biological replicates) of all parasite stages were collected from synchronized infections established in different rats. *Pb*A gametocytes were obtained using a slightly adapted method described by Beetsma *et al.* [41]. Gametocytes were obtained from the reference lines cl15cy1 and 1037cl1 (R). Infected blood containing mature gametocytes was collected from the mice by heart puncture, leucocytes were removed, and gametocytes separated from uninfected cells by Nycodenz gradient centrifugation [13]. Duplicate samples were prepared that were collected from independently infected mice and consisted of 1-2 x 10^8^ gametocytes. In these samples the ratio between male and female gametocytes was approximately 1:1 and samples had <10% contamination with asexual blood stage parasites (cl15cy1: 8%; 1037cl1: 9%; as determined with Giemsa-stained slides of samples). The 24 hour *Pb*A ookinetes (line cl15cy1) were produced using preparations of purified gametocytes that were obtained as described above. 1-2x10^8^ purified gametocytes were incubated in standard ookinete *in vitro* culture for 24h at 21°C as described [42]. In this culture 76% of the female gametocytes transformed into mature ookinetes. For the 16 hour *Pb*A ookinete samples (line 2.34) purified ookinetes were obtained as described [43]. Total RNA isolated in Trizol was chloroform-extracted. Subsequently, RNA was cleaned over an RNeasy Mini column (Qiagen, RNeasy Mini Kit) including genomic DNA digestion by on-column DNase treatment (Qiagen, RNase-free DNase Set). Integrity of RNA samples was confirmed by agarose gel electrophoresis. Further RNA work-up was performed as described previously [44] with few modifications. Briefly, ~2.4 - 7 µg was subjected to selection for PolyA+-RNA (Qiagen, Oligotex mRNA Mini Kit). Subsequently, PolyA+-selected RNA originating from 2.4 - 5 µg total RNA (absolute concentrations are unknown as these low RNA concentrations could not be measured) was fragmented by hydrolysis. Samples were subjected to TURBO DNase treatment (2 units, Ambion) in the presence of 1x NEBuffer 4 (New England Biolabs) in a total volume of 10 µl, for 15 min at 37°C followed by a 10 min inactivation at 70°C. RNA was converted to double-stranded cDNA using AT-corrected random nonamer primers (76% AT) during the first strand synthesis reaction as described [44]. All samples were tested for genomic DNA contamination in a reverse transcriptase minus (RT-) control reaction and were found to be negative. 15-40ng double-stranded cDNA was used for sequencing library preparation as described in [45]. Sequencing libraries were loaded on the Illumina Genome Analyzer IIx and sequenced for 76 cycles from one side of the fragment (Standard Cluster Generation Kit v4 and 2x 36-cycle sequencing kit v4). For the 16 hour *Pb*A ookinete samples RNA was isolated and sequenced as described [43]. Accession numbers for all *Pb*A samples are indicated in **Additional file 17**.

*Pc*AS blood stages (7 day infection; late trophozoite stage, see) were isolated from four BALB/c mice and two C57BL/6 mice as described [46]. mRNA was sequenced using Illumina technology (**Additional file 17;** Array Express accession number: E-ERAD-25). RNA collection, sequencing and further analysis was performed as described [46]. PcAS blood- and vector transmitted samples: Mice (C57BL6) were bled out at 6 days post-infection, and RNA was extracted from purified parasite populations using Trizol reagent and DNase treated. Poly A+ mRNA was purified from total RNA using oligo dT dyna bead selection and libraries were created using a modified RNA-seq protocol, where RNA was fragmented using Covaris AFA sonication instead of metal ions. The samples were sequenced on an Illumina HiSeq 2000 [47] (**Additional file 17;** Array Express accession number: E-ERAD-95). *Py*YM late blood stages of two lines of *Py*YM were collected as described [40] and mRNA was sequenced on an Illumina GA II platform using the Illumina RNA-seq protocol (**Additional file 17;** Array Express accession number: ERS032261, ERS032262). RNA collection, sequencing and further analysis was performed as described [40].
**RNA-seq analysis**
*Mapping/FPKM* values
To correct gene models and to compare the expression between samples, each sample was first mapped against its reference genome using TopHat [48] (version v2.0.6, parameter -g 1). The resulting BAM files were used to detect errors in the gene models and to examine alternative splicing. To determine transcript abundance we calculated the FPKM values for all genes (FPKM: fragments per kilo base of exon per million fragments mapped) using Cufflinks (parameter: -b -u –q [49]. As RNA-seq data can be noisy, an FPKM cut-off value was calculated based on the FPKM of introns. Accepting 10% of the intron as real signal, a cut-off value of 21 was determined over all RNA-seq samples.
*Correlation plots/Heatmaps*
All plots were done in R (Foundation for Statistical Computing; [www.R-project.org](http://www.R-project.org)). The correlation plot was generated with the corrplot function of the corrplot R library. We only included genes that had one-to-one orthologs across the three rodent species. Heatmaps were generated with FPKM for each gene and condition, using the heatmap.2 function of the gplots package. Genes were included that did have a FPKM value >21 in at least one condition/stage. The expression data (FPKM) for each gene was normalised to mean 0 and variance 1, using the “normalize" function of the som R package. The Standard algorithm was chosen for clustering, with the exception of data presented in Figure 3B where the Ward algorithm was used.
*Splice site detection*
To detect errors in the annotation and to find new or alternative splice sites, a custom Perl script was written, which catalogues each RNA-seq read that mapped as a split read (each part of the read must be at least 12bp long). The script measures the number of split reads at specific coordinates in the BAM files. In addition, the script determined whether the read confirmed a putative splice site or generated an alternative splice site.
*Differential expression/GO enrichment*For differential expression cuffdiff [49] (v2.0.2, with parameters -u –q) was used to compensate for GC variation and repetitive regions. GO enrichment was performed in R, using TopGO. As a GO-database, the predicted GO terms from the reference genomes were used (see above). We chose as GO-database the GO-term of all genes with an FPKM > 21 in at least one of the ookinete samples. These were compared with the GO-terms of genes marked as differentially expressed from cuffdiff.
 **Analysis of genes, classification of multigene families** **and phylogenetic analyses
*Orthologous genes***To define orthologous genes between the three rodent genomes and other primate malaria species (*P. falciparum* 3D7, *P. knowlesi* H and *P. vivax* Sall) OrthoMCL [28] was used (version 1.4, standard parameters).
***PEXEL*-motif**
All genes of the reference genomes were analysed for the presence of a PEXEL-motif using the updated HMM algorithm ExportPred v2.0 [50]. As a cutoff value 1.5 was used as in [50]. To compare genes with PEXEL-motifs between the three species we used only orthologous genes with a one-to-one relationship in the 3 RMP species.
***Classification of the multigene families***Multigene families were classified by manual inspection of conserved domains (Interpro) and gene structures.
***Phylogenetic analyses of pirs***All full-length *pir* coding sequences, including predicted pseudogenes, were extracted from the *P. berghei* ANKA (n = 184)*, P. c. chabaudi* AS (n = 193) and *P. y.* *yoelii* YM (n = 783) genome sequences. Translated nucleotide sequences for 1160 genes were aligned in ClustalW [51]; all multiple alignments were then manually edited to ensure that all frame-shifts were resolved. Non-homologous positions at the N-terminus were removed by curtailing the alignment to the N-terminal-most conserved cysteine position. Similarly, non-homologous repetitive motifs were removed from ‘long-form’ PIRs (i.e. 188 proteins > 1200 amino acids in length). The resultant 1266-character alignment constitutes the conserved core of all PIRs and almost the complete expanse of ‘short-forms’ (i.e. <1200 amino acids in length and 972/1160 genes). A Maximum Likelihood (ML) phylogeny was estimated from the nucleotide sequence alignment using RAxML v7.0.4 [52] using a GTR+G model. Node support was assessed using 100 non-parametric bootstrap replicates [53]. A ML phylogeny was also estimated from amino acid sequences using PHYML v3.0 [54] under an LG+G model [55], which produced a tree topology consistent with that generated from nucleotide sequences (data not shown). A Bayesian phylogeny was estimated using MrBayes v3.2.1 [56] with a GTR+G model for a subsample of *pir* nucleotide sequences (MCMC settings: Nruns=4, Ngen=1000000, sample burnin=1000, and default prior distribution). Due to the large number of sequences, it was not possible `to achieve convergence in parameter values during the Bayesian analysis; therefore we selected 135 sequences that represented the various clades observed in the ML tree for a smaller analysis. We observed that the ‘long-form’ pirs comprise a distinct and divergent clade to all other forms; therefore, all trees were rooted using this ‘long-form’ clade as outgroup. The tree was largely not robust: most nodes did not return bootstrap values > 75 or Bayesian posterior probabilities > 0.9. This is particularly true of medium-depth nodes, i.e. those at the base of the labelled clades. However, there were robust nodes meeting these requirements and the molecular systematics has been based around these ‘stable’ features of the tree. There were additional robust nodes towards the tips of the clades relating to close paralogs, but these are not shown. The separation of short-forms from long-forms was robust, in keeping with their structural disparities, even though the alignment did not include the domains unique to the ‘long’ forms; thus the disparity is equally evident from the conserved 3’ end. Branch lengths in the ‘long’ clade are significantly greater than in the ‘short’ clade (p < 0.01; t-test). Defining *pir* subfamilies based on robust nodes in the phylogeny yields eight ‘short’ form clades (S1-8) and four ‘long’ form clades (L1-4). In the case of S1, this clade is not supported by a robust node but it does contain several clades that are well supported (S1a-g). We chose to bring all of these together under a single subfamily with several other sequences that are not robustly placed; otherwise the latter would be unclassified (for example, lineages outside S1d).

**References**

1. Janse CJ, Ramesar J, Waters AP: **High-efficiency transfection and drug selection of genetically transformed blood stages of the rodent malaria parasite Plasmodium berghei.** *Nat Protoc* 2006, **1:**346-356.

2. Pattaradilokrat S, Cheesman SJ, Carter R: **Congenicity and genetic polymorphism in cloned lines derived from a single isolate of a rodent malaria parasite.** *Mol Biochem Parasitol* 2008, **157:**244-247.

3. Beale GH, Carter R., Walliker D.. **Genetics.** In: Rodent Malaria (R. Killick-Kendrick and W. Peters, eds) Academic Press London, 213-246. 1978.

4. Killick-Kendrick R. **Taxonomy, Zoography and Evolution.** In: Rodent Malaria (R. Killick-Kendrick and W. Peters, eds) Academic Press London, 1-52. 1978.

5. Ramiro RS, Reece SE, Obbard DJ: **Molecular evolution and phylogenetics of rodent malaria parasites.** *BMC Evol Biol* 2012, **12:**219.

6. Cheesman S, O'Mahony E, Pattaradilokrat S, Degnan K, Knott S, Carter R: **A single parasite gene determines strain-specific protective immunity against malaria: the role of the merozoite surface protein I.** *Int J Parasitol* 2010, **40:**951-961.

7. Gadsby N, Lawrence R, Carter R: **A study on pathogenicity and mosquito transmission success in the rodent malaria parasite Plasmodium chabaudi adami.** *Int J Parasitol* 2009, **39:**347-354.

8. Pasini EM, Braks JA, Fonager J, Klop O, Aime E, Spaccapelo R, Otto TD, Berriman M, Hiss JA, Thomas AW et al.: **Proteomic and genetic analyses demonstrate that Plasmodium berghei blood stages export a large and diverse repertoire of proteins.** *Mol Cell Proteomics* 2013, **12:**426-448.

9. Janse CJ, Carlton JM, Walliker D, Waters AP: **Conserved location of genes on polymorphic chromosomes of four species of malaria parasites.** *Mol Biochem Parasitol* 1994, **68:**285-296.

10. Vanderberg JP, Nussenzweig RS, Most H: **Further studies on the Plasmodium berghei-Anopheles stephensi--rodent system of mammalian malaria.** *J Parasitol* 1968, **54:**1009-1016.

11. Van den Steen PE, Geurts N, Deroost K, Van A, I, Verhenne S, Heremans H, Van DJ, Opdenakker G: **Immunopathology and dexamethasone therapy in a new model for malaria-associated acute respiratory distress syndrome.** *Am J Respir Crit Care Med* 2010, **181:**957-968.

12. Franke-Fayard B, Janse CJ, Cunha-Rodrigues M, Ramesar J, Buscher P, Que I, Lowik C, Voshol PJ, den Boer MA, van Duinen SG et al.: **Murine malaria parasite sequestration: CD36 is the major receptor, but cerebral pathology is unlinked to sequestration.** *Proc Natl Acad Sci U S A* 2005, **102:**11468-11473.

13. Janse CJ, Waters AP: **Plasmodium berghei: the application of cultivation and purification techniques to molecular studies of malaria parasites.** *Parasitol Today* 1995, **11:**138-143.

14. Trang DT, Huy NT, Kariu T, Tajima K, Kamei K: **One-step concentration of malarial parasite-infected red blood cells and removal of contaminating white blood cells.** *Malar J* 2004, **3:**7.

15. Spence PJ, Cunningham D, Jarra W, Lawton J, Langhorne J, Thompson J: **Transformation of the rodent malaria parasite Plasmodium chabaudi.** *Nat Protoc* 2011, **6:**553-561.

16. Chain PS, Grafham DV, Fulton RS, Fitzgerald MG, Hostetler J, Muzny D, Ali J, Birren B, Bruce DC, Buhay C et al.: **Genomics. Genome project standards in a new era of sequencing.** *Science* 2009, **326:**236-237.

17. Kozarewa I, Ning Z, Quail MA, Sanders MJ, Berriman M, Turner DJ: **Amplification-free Illumina sequencing-library preparation facilitates improved mapping and assembly of (G+C)-biased genomes.** *Nat Methods* 2009, **6:**291-295.

18. Mullikin JC, Ning Z: **The phusion assembler.** *Genome Res* 2003, **13:**81-90.

19. Otto TD, Sanders M, Berriman M, Newbold C: **Iterative Correction of Reference Nucleotides (iCORN) using second generation sequencing technology.** *Bioinformatics* 2010, **26:**1704-1707.

20. Zerbino DR, Birney E: **Velvet: algorithms for de novo short read assembly using de Bruijn graphs.** *Genome Res* 2008, **18:**821-829.

21. Assefa S, Keane TM, Otto TD, Newbold C, Berriman M: **ABACAS: algorithm-based automatic contiguation of assembled sequences.** *Bioinformatics* 2009, **25:**1968-1969.

22. Tsai IJ, Otto TD, Berriman M: **Improving draft assemblies by iterative mapping and assembly of short reads to eliminate gaps.** *Genome Biol* 2010, **11:**R41.

23. Boetzer M, Henkel CV, Jansen HJ, Butler D, Pirovano W: **Scaffolding pre-assembled contigs using SSPACE.** *Bioinformatics* 2011, **27:**578-579.

24. Carver T, Berriman M, Tivey A, Patel C, Bohme U, Barrell BG, Parkhill J, Rajandream MA: **Artemis and ACT: viewing, annotating and comparing sequences stored in a relational database.** *Bioinformatics* 2008, **24:**2672-2676.

25. Otto TD, Dillon GP, Degrave WS, Berriman M: **RATT: Rapid Annotation Transfer Tool.** *Nucleic Acids Res* 2011, **39:**e57.

26. Hunter S, Apweiler R, Attwood TK, Bairoch A, Bateman A, Binns D, Bork P, Das U, Daugherty L, Duquenne L et al.: **InterPro: the integrative protein signature database.** *Nucleic Acids Res* 2009, **37:**D211-D215.

27. Hall N, Karras M, Raine JD, Carlton JM, Kooij TW, Berriman M, Florens L, Janssen CS, Pain A, Christophides GK et al.: **A comprehensive survey of the Plasmodium life cycle by genomic, transcriptomic, and proteomic analyses.** *Science* 2005, **307:**82-86.

28. Li L, Stoeckert CJ, Jr., Roos DS: **OrthoMCL: identification of ortholog groups for eukaryotic genomes.** *Genome Res* 2003, **13:**2178-2189.

29. Boetzer M, Pirovano W: **Toward almost closed genomes with GapFiller.** *Genome Biol* 2012, **13:**R56.

30. Hunt M, Kikuchi T, Sanders M, Newbold C, Berriman M, Otto TD: **REAPR: a universal tool for genome assembly evaluation.** *Genome Biol* 2013, **14:**R47.

31. Simpson JT, Durbin R: **Efficient de novo assembly of large genomes using compressed data structures.** *Genome Res* 2012, **22:**549-556.

32. Swain MT, Tsai IJ, Assefa SA, Newbold C, Berriman M, Otto TD: **A post-assembly genome-improvement toolkit (PAGIT) to obtain annotated genomes from contigs.** *Nat Protoc* 2012, **7:**1260-1284.

33. Stanke M, Steinkamp R, Waack S, Morgenstern B: **AUGUSTUS: a web server for gene finding in eukaryotes.** *Nucleic Acids Res* 2004, **32:**W309-W312.

34. McKenna A, Hanna M, Banks E, Sivachenko A, Cibulskis K, Kernytsky A, Garimella K, Altshuler D, Gabriel S, Daly M et al.: **The Genome Analysis Toolkit: a MapReduce framework for analyzing next-generation DNA sequencing data.** *Genome Res* 2010, **20:**1297-1303.

35. Li H, Handsaker B, Wysoker A, Fennell T, Ruan J, Homer N, Marth G, Abecasis G, Durbin R: **The Sequence Alignment/Map format and SAMtools.** *Bioinformatics* 2009, **25:**2078-2079.

36. Morgulis A, Gertz EM, Schaffer AA, Agarwala R: **A fast and symmetric DUST implementation to mask low-complexity DNA sequences.** *J Comput Biol* 2006, **13:**1028-1040.

37. Billker O, Dechamps S, Tewari R, Wenig G, Franke-Fayard B, Brinkmann V: **Calcium and a calcium-dependent protein kinase regulate gamete formation and mosquito transmission in a malaria parasite.** *Cell* 2004, **117:**503-514.

38. Spaccapelo R, Janse CJ, Caterbi S, Franke-Fayard B, Bonilla JA, Syphard LM, Di CM, Dottorini T, Savarino A, Cassone A et al.: **Plasmepsin 4-deficient Plasmodium berghei are virulence attenuated and induce protective immunity against experimental malaria.** *Am J Pathol* 2010, **176:**205-217.

39. Slade SJ, Langhorne J: **Production of interferon-gamma during infection of mice with Plasmodium chabaudi chabaudi.** *Immunobiology* 1989, **179:**353-365.

40. Ogun SA, Tewari R, Otto TD, Howell SA, Knuepfer E, Cunningham DA, Xu Z, Pain A, Holder AA: **Targeted disruption of py235ebp-1: invasion of erythrocytes by Plasmodium yoelii using an alternative Py235 erythrocyte binding protein.** *PLoS Pathog* 2011, **7:**e1001288.

41. Beetsma AL, van de Wiel TJ, Sauerwein RW, Eling WM: **Plasmodium berghei ANKA: purification of large numbers of infectious gametocytes.** *Exp Parasitol* 1998, **88:**69-72.

42. Janse CJ, Mons B, Rouwenhorst RJ, Van der Klooster PF, Overdulve JP, Van der Kaay HJ: **In vitro formation of ookinetes and functional maturity of Plasmodium berghei gametocytes.** *Parasitology* 1985, **91 ( Pt 1):**19-29.

43. Sebastian S, Brochet M, Collins MO, Schwach F, Jones ML, Goulding D, Rayner JC, Choudhary JS, Billker O: **A Plasmodium calcium-dependent protein kinase controls zygote development and transmission by translationally activating repressed mRNAs.** *Cell Host Microbe* 2012, **12:**9-19.

44. Bartfai R, Hoeijmakers WA, Salcedo-Amaya AM, Smits AH, Janssen-Megens E, Kaan A, Treeck M, Gilberger TW, Francoijs KJ, Stunnenberg HG: **H2A.Z demarcates intergenic regions of the plasmodium falciparum epigenome that are dynamically marked by H3K9ac and H3K4me3.** *PLoS Pathog* 2010, **6:**e1001223.

45. Hoeijmakers WA, Bartfai R, Francoijs KJ, Stunnenberg HG: **Linear amplification for deep sequencing.** *Nat Protoc* 2011, **6:**1026-1036.

46. Lawton J, Brugat T, Yam XY, Reid AJ, Boehme U, Otto TD, Pain A, Jackson A, Berriman M, Cunningham D et al.: **Characterization and gene expression analysis of the cir multi-gene family of Plasmodium chabaudi chabaudi (AS).** *BMC Genomics* 2012, **13:**125.

47. Spence PJ, Jarra W, Levy P, Reid AJ, Chappell L, Brugat T, Sanders M, Berriman M, Langhorne J: **Vector transmission regulates immune control of Plasmodium virulence.** *Nature* 2013, **498:**228-231.

48. Trapnell C, Pachter L, Salzberg SL: **TopHat: discovering splice junctions with RNA-Seq.** *Bioinformatics* 2009, **25:**1105-1111.

49. Trapnell C, Hendrickson DG, Sauvageau M, Goff L, Rinn JL, Pachter L: **Differential analysis of gene regulation at transcript resolution with RNA-seq.** *Nat Biotechnol* 2013, **31:**46-53.

50. Boddey JA, Carvalho TG, Hodder AN, Sargeant TJ, Sleebs BE, Marapana D, Lopaticki S, Nebl T, Cowman AF: **Role of Plasmepsin V in Export of Diverse Protein Families from the Plasmodium falciparum Exportome.** *Traffic* 2013, **14:**532-550.

51. Larkin MA, Blackshields G, Brown NP, Chenna R, McGettigan PA, McWilliam H, Valentin F, Wallace IM, Wilm A, Lopez R et al.: **Clustal W and Clustal X version 2.0.** *Bioinformatics* 2007, **23:**2947-2948.

52. Stamatakis A, Ludwig T, Meier H: **RAxML-III: a fast program for maximum likelihood-based inference of large phylogenetic trees.** *Bioinformatics* 2005, **21:**456-463.

53. Stamatakis A, Hoover P, Rougemont J: **A rapid bootstrap algorithm for the RAxML Web servers.** *Syst Biol* 2008, **57:**758-771.

54. Guindon S, Gascuel O: **A simple, fast, and accurate algorithm to estimate large phylogenies by maximum likelihood.** *Syst Biol* 2003, **52:**696-704.

55. Le SQ, Gascuel O: **An improved general amino acid replacement matrix.** *Mol Biol Evol* 2008, **25:**1307-1320.

56. Ronquist F, Huelsenbeck JP: **MrBayes 3: Bayesian phylogenetic inference under mixed models.** *Bioinformatics* 2003, **19:**1572-1574.
