# Supplementary material for: A comprehensive evaluation of rodent malaria parasite genomes and gene expression
Source: BMC Biol. 2014 Oct 30;12:86. doi: 10.1186/s12915-014-0086-0 (PMC4242472; doi:10.1186/s12915-014-0086-0)
Supplement: Additional file 12: — Distribution of members of different multigene families on chromosomes of Pb A. [file 12915_2014_86_MOESM12_ESM.pdf]

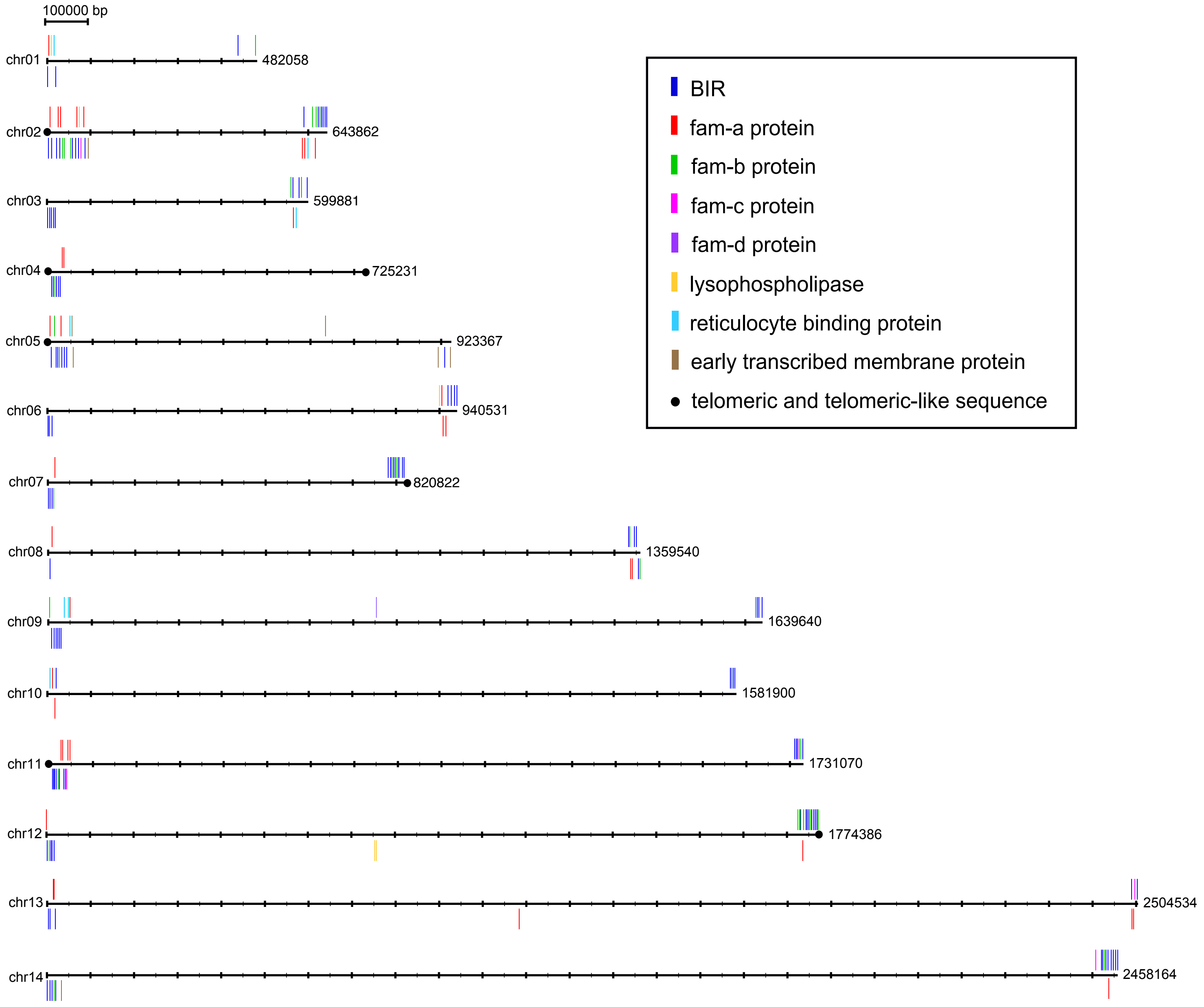

Figure S2
Distribution of members of different multigene families on chromosomes of *Pb*A (related to Figure 1).
