# Supplementary material for: A comprehensive evaluation of rodent malaria parasite genomes and gene expression
Source: BMC Biol. 2014 Oct 30;12:86. doi: 10.1186/s12915-014-0086-0 (PMC4242472; doi:10.1186/s12915-014-0086-0)
Supplement: Additional file 13: — Distribution of members of different multigene families on chromosomes of Pc AS. [file 12915_2014_86_MOESM13_ESM.pdf]

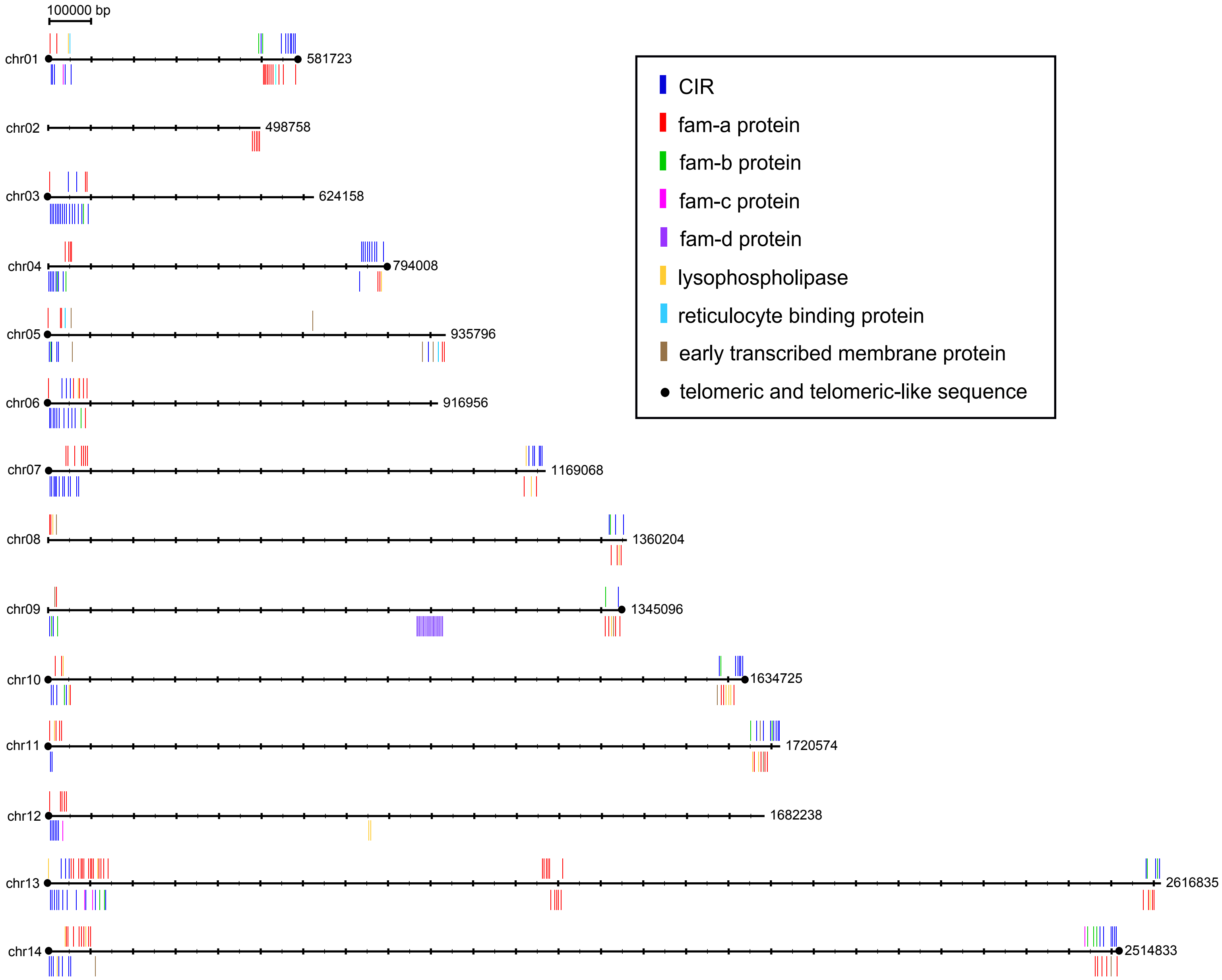

Figure S3
Distribution of members of different multigene families on chromosomes of *Pc*AS (related to Figure 1).
