# Supplementary material for: A comprehensive evaluation of rodent malaria parasite genomes and gene expression
Source: BMC Biol. 2014 Oct 30;12:86. doi: 10.1186/s12915-014-0086-0 (PMC4242472; doi:10.1186/s12915-014-0086-0)
Supplement: Additional file 16: — Expression of PIRs in relation to their phylogenetic relationship. Heat maps of expression (FPKM values >21; normalized by gene) of all PbA pirs in different life cycle stages in association with their location in different clades (L, S) of the phylogenetic tree (black boxes). Ri: ring; Tr: Trophozoite; Sch: Schizont; Gct: Gametocyte; Ook: Ookinetes (16 and 24 hour ookinetes). [file 12915_2014_86_MOESM16_ESM.pdf]

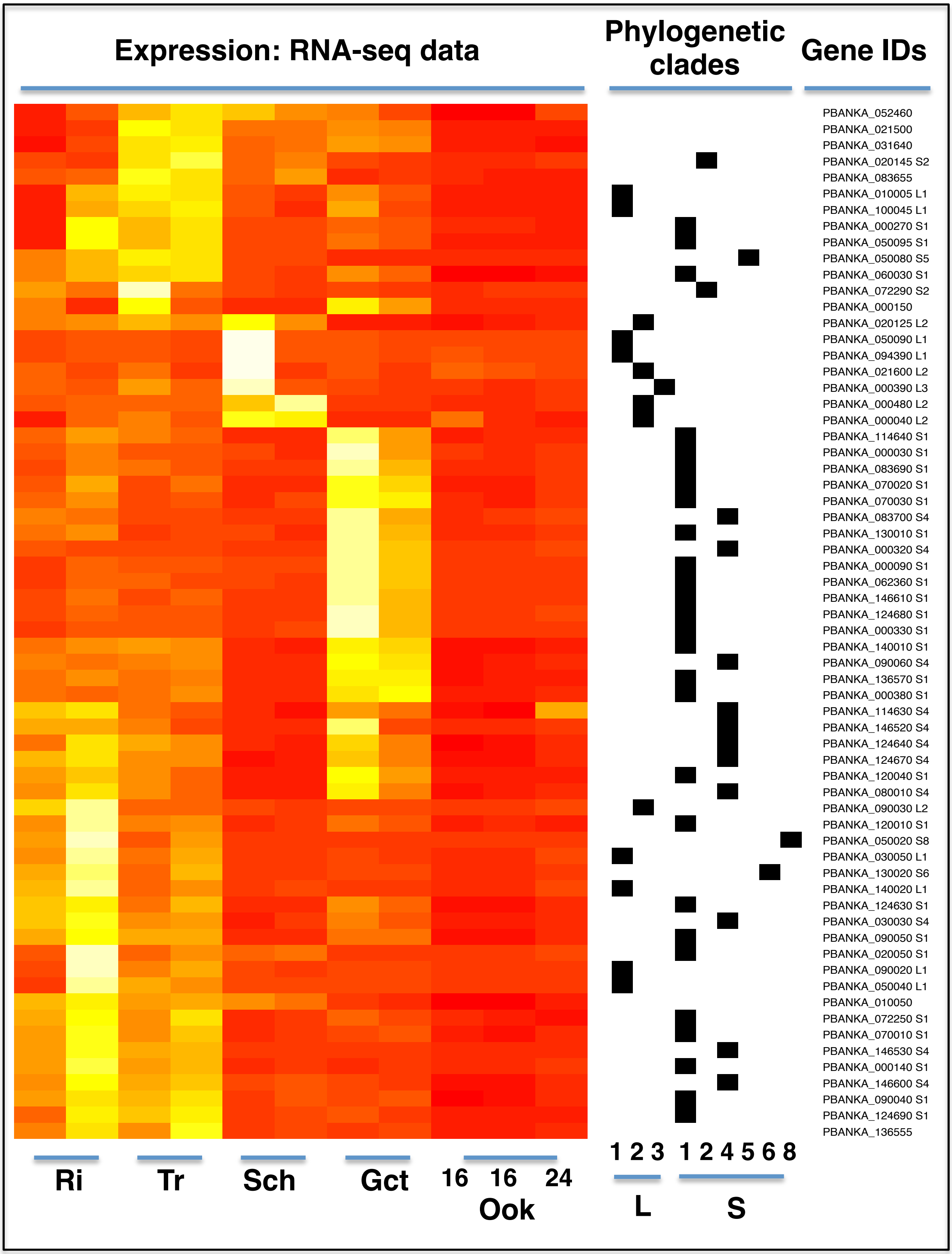

Figure S5

Expression of PIRs in relation to their phylogenetic relationship. Heat maps of expression (FPKM values >21; normalized by gene) of all *Pb*A *pirs* in different life cycle stages in association with their location in different clades (L, S) of the phylogenetic tree (black boxes). Ri: ring; Tr: Trophozoite; Sch: Schizont; Gct: Gametocyte; Ook: Ookinetes (16 and 24 hour ookinetes)(related to Figure 4).
